# Epigenetic Regulation of Vascular Smooth Muscle Cells by Histone H3 Lysine 9 Dimethylation Attenuates Target Gene-Induction by Inflammatory Signaling

**DOI:** 10.1161/ATVBAHA.119.312765

**Published:** 2019-08-22

**Authors:** Jennifer L. Harman, Lina Dobnikar, Joel Chappell, Benjamin G. Stokell, Amanda Dalby, Kirsty Foote, Alison Finigan, Paula Freire-Pritchett, Annabel L. Taylor, Matthew D. Worssam, Ralitsa R. Madsen, Elena Loche, Anna Uryga, Martin R. Bennett, Helle F. Jørgensen

**Affiliations:** 1 From the Division of Cardiovascular Medicine, Department of Medicine, University of Cambridge, United Kingdom (J.L.H., L.D., J.C., A.D., K.F., A.F., A.L.T., M.D.W., R.R.M., E.L., A.U., M.R.B., H.F.J.); 2 Babraham Institute, Babraham Research Campus, Cambridge, United Kingdom (L.D., P.F.-P.); 3 Statistical Laboratory, Centre for Mathematical Sciences, University of Cambridge, United Kingdom (B.G.S.).

**Keywords:** arteries, chromatin immunoprecipitation, cytokines, epigenetics, histone, inflammation

## Abstract

Vascular inflammation underlies cardiovascular disease. Vascular smooth muscle cells (VSMCs) upregulate selective genes, including MMPs (matrix metalloproteinases) and proinflammatory cytokines upon local inflammation, which directly contribute to vascular disease and adverse clinical outcome. Identification of factors controlling VSMC responses to inflammation is therefore of considerable therapeutic importance. Here, we determine the role of Histone H3 lysine 9 di-methylation (H3K9me2), a repressive epigenetic mark that is reduced in atherosclerotic lesions, in regulating the VSMC inflammatory response.

**Approach and Results:**

We used VSMC-lineage tracing to reveal reduced H3K9me2 levels in VSMCs of arteries after injury and in atherosclerotic lesions compared with control vessels. Intriguingly, chromatin immunoprecipitation showed H3K9me2 enrichment at a subset of inflammation-responsive gene promoters, including *MMP3*, *MMP9*, *MMP12*, and *IL6*, in mouse and human VSMCs. Inhibition of G9A/GLP (G9A-like protein), the primary enzymes responsible for H3K9me2, significantly potentiated inflammation-induced gene induction in vitro and in vivo without altering NFκB (nuclear factor kappa-light-chain-enhancer of activated B cell) and MAPK (mitogen-activated protein kinase) signaling. Rather, reduced G9A/GLP activity enhanced inflammation-induced binding of transcription factors NFκB-p65 and cJUN to H3K9me2 target gene promoters *MMP3* and *IL6*. Taken together, these results suggest that promoter-associated H3K9me2 directly attenuates the induction of target genes in response to inflammation in human VSMCs.

**Conclusions:**

This study implicates H3K9me2 in regulating the proinflammatory VSMC phenotype. Our findings suggest that reduced H3K9me2 in disease enhance binding of NFκB and AP-1 (activator protein-1) transcription factors at specific inflammation-responsive genes to augment proinflammatory stimuli in VSMC. Therefore, H3K9me2-regulation could be targeted clinically to limit expression of MMPs and IL6, which are induced in vascular disease.

## Abstract 2

Supplemental Digital Content is available in the text.

**Highlights**

- The repressive epigenetic mark Histone H3 Lysine 9 Dimethylation (H3K9me2) is reduced in vascular smooth muscle cells on inflammation in cardiovascular disease models.
- Loss of H3K9me2 in human and mouse vascular smooth muscle cells exacerbates inflammation-induced upregulation of disease-associated genes in vitro and in vivo, with functional consequences for matrix degradation and phenotypic switching.
- H3K9me2 inhibits binding of NFκB (nuclear factor kappa-light-chain-enhancer of activated B cell) and AP-1 (activator protein-1) transcription factors to their cognate sequences within MMP (matrix metalloproteinase) and cytokine gene promoters.

**See accompanying editorial on page 2199**

Vascular smooth muscle cell (VSMC) accumulation is a hallmark of cardiovascular disease (CVD), including atherosclerosis and vascular remodeling after injury. In response to proinflammatory stimuli, VSMCs downregulate contractile proteins and upregulate selective gene sets, including matrix metalloproteinases, and proinflammatory cytokines (eg, IL [interleukin]-6), which directly contribute to the progression of vascular disease.^1,2^ Inflammation-responsive gene expression is controlled by the NFκB (nuclear factor kappa-light-chain-enhancer of activated B cells) and AP-1 (activator protein 1) transcription factors, and dysregulation of NFκB and MAPK (mitogen-activated protein kinase)/AP-1 signaling is linked to the initiation and progression of vascular dysfunction.^3^ Indeed, directly reducing inflammation with Canakinumab, an IL1-β neutralizing antibody, significantly lowered cardiovascular events compared with placebo in patients^4^ although not in mice.^5^ Identification of mechanisms that regulate inflammation-associated changes in VSMC gene expression is therefore of considerable therapeutic importance.

MMPs (matrix metalloproteinases) comprise a family of proteases that degrade extracellular matrix components and mediate many structural changes associated with vascular disease.^6,7^ For example, animal atherosclerosis studies implicate MMP12 in plaque development and instability.^8,9^ MMP3 and MMP9 promote VSMC migration and neointima formation after carotid ligation in mice.^10^ In addition, increased MMP3, MMP9, and MMP12 activity is observed in vulnerable compared with stable human atherosclerotic lesions.^11–14^ The proinflammatory cytokine IL-6 also plays an important role in vascular disease progression. IL-6 attenuates VSMC contractility while promoting VSMC migration, proliferation, and vascular calcification.^15–17^ Furthermore, elevated serum IL-6 is associated with greater CVD risk.^18–20^

Gene expression is regulated by epigenetic pathways that modify histone proteins, resulting in either increased or reduced accessibility to the transcription machinery.^21^ Decreased levels of histone H3 lysine 9 di-methylation (H3K9me2) and histone H3 lysine 27 tri-methylation (H3K27me3) are observed in VSMCs derived from human atherosclerotic plaques compared with control arteries.^22^ However, how these mechanisms control inflammation-associated changes in VSMC gene expression are unclear. Interestingly, H3K9me2, a repressive mark associated with facultative heterochromatin^23^ and directly regulating the expression of inducible genes in other cells,^24–28^ affects the growth rate, migration, and contractility of pulmonary and airway smooth muscle cells.^29,30^ Yet, the functional importance and target genes of H3K9me2 in VSMCs remain largely unknown.

We demonstrate that global levels of H3K9me2 in VSMCs are reduced in atherosclerosis and in arteries undergoing injury-induced remodeling, concomitant with increased inflammation. H3K9me2 is deposited at a subset of inflammation-responsive VSMC genes, including *MMP3*, *MMP9*, *MMP12*, and *IL6*, which are strongly associated with CVD.^11–14^ Importantly, we show that H3K9me2 attenuates inflammation-associated upregulation of target genes by inhibiting NFκB/p65 and/or AP-1/cJUN transcription factor binding. These findings identify functional consequences of loss of H3K9me2 in vascular disease and suggest H3K9me2 as an important mechanism to prevent spurious induction of a proinflammatory state in VSMCs.

## Methods

### Disclosure Statement

The chromatin immunoprecipitation (ChIP)-seq data have been made publicly available at the Gene Expression Omnibus with the accession number GSE131212. All other supporting data are available within the article and in the online-only Data Supplement.

### Animal Experiments

All experiments were approved by the United Kingdom Home Office (PPL70/7565 and P452C9545) and the local ethics committee. Wild-type and Apolipoprotein E-null (*Apoe*^−/−^) mice on a C57Bl/6 background were purchased from Charles River. *Myh11-Cre*^*ERt2*^ (Y-linked),^31^ *Rosa26-Confetti*, *Rosa26-EYFP* (enhanced yellow fluorescent protein), and *Apoe*^−/−^ mice have been described previously.^31,32^ MYH11 is a marker of differentiated smooth muscle cells and the *Myh11-Cre*^*ERt2*^ transgene used here has been extensively tested for VSMC-specific expression in major arteries.^31,33–36^ Experimental animals (all males as the *Myh11-Cre*^*ERt2*^ transgene is Y-linked) received 10 intraperitoneal injections of 1 mg/mL tamoxifen between 4 and 8 weeks of age for lineage labeling. To inhibit G9A/GLP (G9A-like protein), lineage-labeled *Myh11-Cre*^*ERt2*^*/Rosa26-EYFP+* males were treated with A366 delivered by osmotic pumps (30 mg/kg per day in 98:2 PEG 400/polysorbate 80), or vehicle alone, and either analyzed directly or subjected to ligation of the left common carotid artery as described previously.^34^ Surgery and tissue processing are described in the Methods in the online-only Data Supplement.

### ChIP and Analysis

ChIP was performed as described in the Methods in the online-only Data Supplement. For genome-wide analysis, the DNA SMART ChIP-seq kit (Clontech, 634865) was used to generate Illumina-compatible sequencing libraries from 100 pg to 2 ng of DNA from 2 independent H3K9me2 ChIP experiments and associated input. Libraries were sequenced (Illumina NextSeq) using paired-end 75 bp reads. ChIP-seq reads were trimmed using Cutadapt v1.9,^37^ aligned to the mouse GRCm38 genome using Bowtie2 v2.2,^38^ and reads per gene promoter (within ±1 kb of the transcription start sites) quantified using SeqMonk v1.4 (http://www.bioinformatics.babraham.ac.uk/projects/seqmonk). Genes showing fewer than 20 read counts in the input samples were removed from further analysis and the ratio of H3K9me2/input signal was computed. Of genes with H3K9me2/input ratios in the top 25th percentile, 63 genes were associated with arteriosclerosis according to the Cardiovascular Disease Portal^39^ (https://rgd.mcw.edu/rgdCuration/?module=portal&func=show&name=cardio).

### VSMC Culture

Human aortic VSMCs (hVSMCs) were isolated as described^40^ from patients undergoing aortic valve replacement with ethics committee approval and used at passage 6 to 15. Primary mouse aortic VSMCs were derived from 8- to 12-week-old wild-type C57Bl/6 males and used at passage 4. VSMCs were treated with UNC0638 (UNC; 1 μM Tocris), small interfering RNA targeting G9A (sc-43777, Santa Cruz), control small interfering RNA (sc-37007, Santa Cruz), SP600125 (10 μM, Abcam), human recombinant IL-1α (2 ng/mL, Peprotech), and human recombinant TNF-α (tumor necrosis factor-α), 90 ng/mL, Peprotech as indicated and analyzed as described in the Methods in the online-only Data Supplement.

### Statistics

Data are shown as mean±SEM, unless otherwise indicated. The number of animals per group and number of biologically independent experiments are indicated in Figure legends (at least 3). Independent in vitro experiment were done using isolates derived from different individuals for hVSMCs and independent primary cultures for murine VSMCs. Data were analyzed using Mann-Whitney *U* test, Kruskal-Wallis 1-way ANOVA with Dunn test to compare specific sample pairs, 1-sided exact Wilcoxon rank-sum tests or a linear model (described in the online-only Data Supplement) with *P*<0.05 as the threshold for considering results to be statistically significant.

## Results

### H3K9me2 Is Reduced Within VSMCs in Atherosclerosis and Arterial Remodeling, Concomitant With Inflammation

VSMCs downregulate lineage markers in atherosclerosis and after injury, making specific quantification of H3K9me2 in VSMCs difficult. We therefore used genetic lineage tracing to definitively identify VSMCs by crossing *Myh11-Cre*^*ERt2+*^ mice^31^ with recombination reporter alleles (Rosa26-Confetti^32^ or Rosa26-EYFP^33^). Tamoxifen treatment of these animals induces VSMC-specific recombination and stable fluorescent protein expression in 70% to 95% of VSMCs in Confetti^34^ and 40% to 60% in EYFP reporter mice.^33,35^ Importantly, the fluorescent lineage reporters are stably expressed, independent of the expression status of the *Myh11-Cre*^*ERt2*^ transgene, in medial VSMCs and in VSMC-derived neo-intimal and plaque cells.^34^

**Figure 1.**
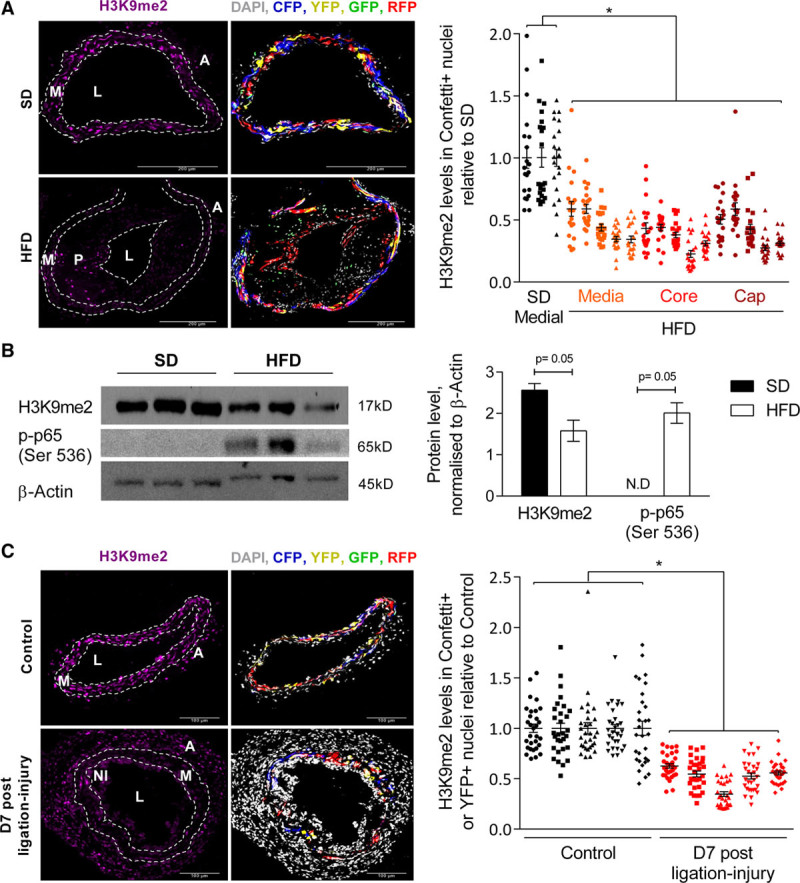
**Histone H3 lysine 9 dimethylation (H3K9me2) is reduced in vascular smooth muscle cells (VSMCs) in atherosclerosis and vascular remodeling.** **A**, Representative immunofluorescence images and quantification of H3K9me2 signal intensity in nuclei of Confetti^+^ cells within left common carotid arteries (LCCAs) from lineage-labeled *Myh11-Cre*^*ERt2+*^*/Rosa26-Confetti*^*+*^*/Apoe*^−/−^ males fed a standard (SD, n=3 animals) or high-fat diet (HFD, n=5 animals). H3K9me2 quantification is displayed separately for medial, plaque core, and cap VSMCs in HFD mice. **B**, Western blot and quantification of H3K9me2 and p-p65 (Ser 536) in aortic media from SD and HFD animals, normalized to β-Actin; n=3 animals per group. N.D., not detected. *P* were calculated as described in the online-only Data Supplement. **C**, Representative immunofluorescence images and quantification of H3K9me2 signal intensity in nuclei of Confetti^+^ cells within LCCAs from ligated (7 days post-ligation) relative to no surgery control *Myh11-Cre*^*ERt2+*^*/Rosa26-Confetti*^*+*^ mice. n=5 animals per group. **A** and **C**, Signals for H3K9me2 (magenta), Confetti reporter proteins (red, blue, yellow, green) and DAPI (4′,6-diamidino-2-phenylindole; white) are shown. The dot plots show H3K9me2 intensity in individual nuclei (of Confetti^+^ cells) relative to the average H3K9me2 signal intensity in control animals (SD in **A**, nonligated control samples in **C**) analyzed in the same batch (batches are indicated with symbols). Mean (line) and SEM (error bars) are indicated. *P<0.05 (linear model, see online-only Data Supplement). A indicates adventitia; L, lumen; M, media; NI, neointima; and P, plaque.

We first crossed *Myh11-Cre*^*ERt2*^*/Rosa26-Confetti* mice with *Apoe*^−/−^ mice to assess H3K9me2 levels in atherosclerosis. Male mice received tamoxifen and were then fed an atherosclerosis-inducing high-fat diet (21% fat, 0.2% cholesterol) or a standard chow diet. Immunohistochemistry in carotid artery sections of animals after 3 to 4 months of high-fat diet revealed significantly lower H3K9me2 levels in Confetti^+^ cells within the media, core, and fibrous cap in atherosclerotic arteries compared with cells in control vessels (Figure 1A). Western blot analysis also indicated reduced H3K9me2 expression in aortas of mice fed a high-fat diet for 6 months compared with control aortas that had little or no visible plaque, and this was associated with increased levels of phosphorylated NFκB p65 (Figure 1B). This indicates that the previously reported reduced H3K9me2 levels in human atherosclerotic plaques compared with healthy controls^22^ is because of downregulation in VSMCs.

To assess whether H3K9me2 regulates early events in the VSMC inflammatory response, we performed ligation of the left common carotid artery, which elicits reproducible vessel remodeling concomitant with changes in VSMC gene expression, including induction of IL6 and MMPs.^41,42^ Immunostaining revealed significantly decreased H3K9me2 levels in lineage-labeled VSMC nuclei in ligated compared with nonligated control arteries at 5, 7, and 28 days post-ligation (Figure 1C and Figure I in the online-only Data Supplement), suggesting that H3K9me2 may regulate the inflammatory response of VSMCs.

### H3K9me2 Attenuates Inflammation-Induced MMP Gene Induction

**Figure 2.**
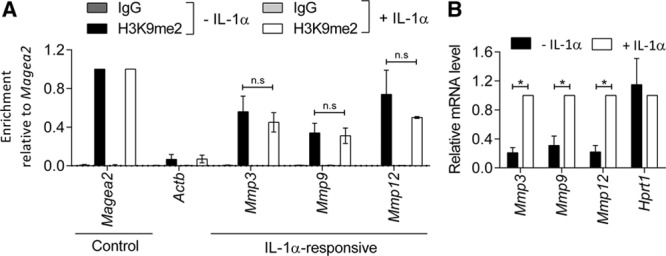
**Histone H3 Lysine 9 Dimethylation (H3K9me2) is enriched at a subset of IL (interleukin)-1α−responsive gene promoters.** **A**, Chromatin immunoprecipitation-qPCR analysis for H3K9me2 in untreated or IL-1α-treated murine vascular smooth muscle cells (mVSMCs; 6 h). H3K9me2 enrichment (relative to *Magea2*) at *Actb* (negative control) and the promoters of *Mmp3*, *Mmp9*, and *Mmp12*, compared with signal observed using negative control IgG (immunoglobulin). Graph show mean±SEM of 3 independent primary cultures. n.s., not significant (Kruskal-Wallis). **B**, RT-qPCR (reverse transcription with quantitative polymerase chain reaction) analysis of *Mmp3*, *Mmp9*, *Mmp12*, and *Hprt1* in control and IL-1α-treated mVSMCs. Expression (mean±SEM in 6 independent primary cultures) is shown relative to IL-1α-treated mVSMCs, normalized to housekeeping genes (*Hmbs and Hprt1*). *P<0.05 (2-tailed Mann-Whitney *U* test).

The reduction of H3K9me2 in VSMCs concomitant with increased inflammation prompted us to examine local levels of H3K9me2 at promoters of arteriosclerosis-associated genes. Genome-wide mapping by ChIP-seq demonstrated that H3K9me2 levels at gene promoters correlated negatively with expression of the associated gene in ex vivo murine VSMCs (Figure II in the online-only Data Supplement), which is expected for a repressive epigenetic mark. We found that among the genes with high H3K9me2 levels within ±1kb of their transcription start site were 63 genes listed as arteriosclerosis-associated on the Cardiovascular Disease Portal^39^ (Table I in the online-only Data Supplement), including several inflammation-responsive MMPs, such as *Mmp3*, *Mmp9*, and *Mmp12.* ChIP-qPCR using cultured primary murine VSMCs confirmed abundant H3K9me2 levels at *Mmp3*, *Mmp9*, and *Mmp12* as well as the positive control locus *Magea2* compared with the negative control, *Actb* (Figure 2A). Surprisingly, despite increased mRNA expression of *Mmp3*, *Mmp9*, and *Mmp12* after IL-1α treatment (Figure 2B), the levels of the repressive H3K9me2 modification at the *Mmp3*, *Mmp9*, and *Mmp12* promoters were not reduced after IL-1α treatment (Figure 2A). The continued presence of this repressive epigenetic mark at target genes suggests that their activation in response to inflammation could be obstructed.

**Figure 3.**
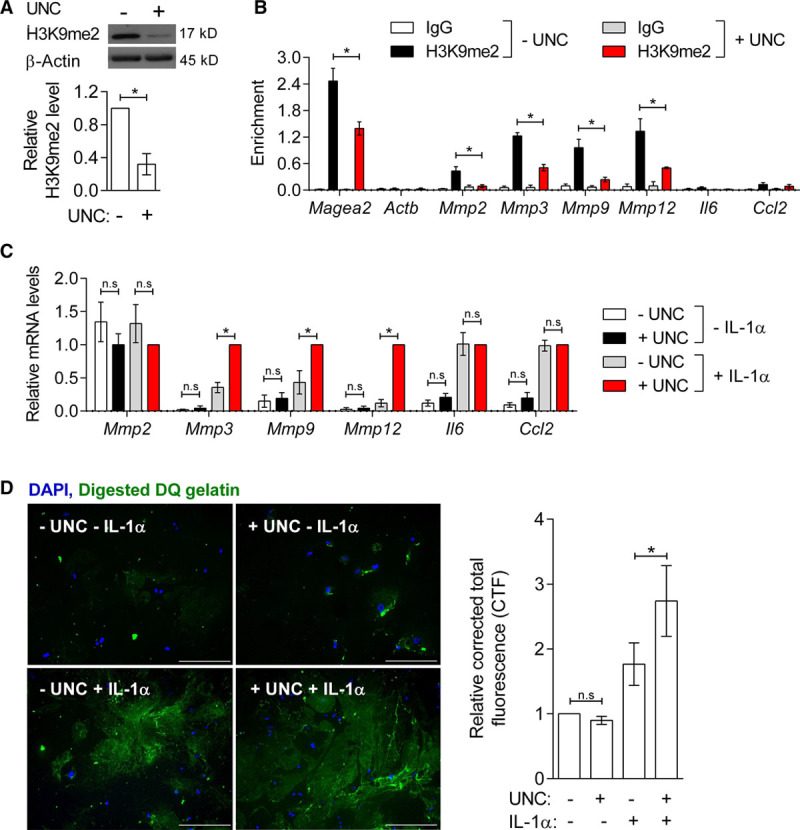
**Inhibition of G9A/GLP (G9A-like protein) reduces Histone H3 Lysine 9 Dimethylation (H3K9me2) at MMP (matrix metalloproteinase) gene promoters and potentiates their upregulation in response to IL-1α.** **A**, Representative western blot and quantification of H3K9me2 in UNC (UNC0638)-treated murine vascular smooth muscle cells (mVSMCs; 48 h) relative to controls (–UNC), normalized to β-actin. Graph shows mean±SEM of 4 independent primary cultures. *P<0.05 (Mann-Whitney *U* test). **B**, Chromatin immunoprecipitation-qPCR analysis in control and UNC-treated mVSMCs showing H3K9me2 enrichment (mean±SEM of 4 independent primary cultures) at control loci (*Magea2*, positive and *Actb*, negative) and the promoters of *Mmp2*, *Mmp3*, *Mmp9*, *Mmp12*, *Il6*, and *Ccl2* compared with negative control IgG. *P<0.05 (Kruskal-Wallis). **C**, RT-qPCR (reverse transcription with quantitative polymerase chain reaction) analysis of *Mmp2*, *Mmp3*, *Mmp9*, *Mmp12*, *Il6*, and *Ccl2* in control (white bars), UNC (black), IL-1α (gray), and IL-1α+UNC-treated (red) mVSMCs. Expression (mean±SEM of 4–5 independent primary cultures) is shown relative to cells treated with UNC+IL-1α (red bars) and normalized to housekeeping genes (*Hmbs* and *Ywhaz*). *P<0.05, n.s., not significant (Kruskal-Wallis). **D**, Representative images and quantification of dye-quenched (DQ)-gelatin digestion in control (top left), UNC (top right), IL-1α (lower left), and UNC+IL-1α-treated mVSMCs (lower right). Signals for digested DQ-gelatin (green) and DAPI (4′,6-diamidino-2-phenylindole)-stained nuclei (blue) are shown. Scale bars, 25 μm. Bar plot shows the corrected total fluorescence (CTF), relative to untreated cells (mean±SEM of 6 independent primary cultures). *P<0.05 (Kruskal-Wallis).

To directly test the importance of H3K9me2-marking at MMP gene promoters, we pretreated cultured murine VSMCs with UNC, an inhibitor of the main H3K9 dimethyltransferases G9A/GLP, before IL-1α stimulation. UNC treatment significantly reduced H3K9me2 levels globally and at target gene promoters (Figure 3A and 3B). UNC did not affect basal MMP gene expression, but significantly potentiated IL-1α-mediated upregulation of *Mmp3* (3.2-fold), *Mmp9* (1.7-fold), and *Mmp12* (7.1-fold) (Figure 3C). In contrast, neither IL-1α nor UNC treatment alone, or in combination, affected the expression of *Mmp2*, which is constitutively expressed by VSMCs.^7^ Importantly, UNC+IL-1α did not affect expression of *Il6* and *Ccl2* (Figure 3C), which showed no H3K9me2 enrichment (Figure 3B), demonstrating that the effect of UNC on MMP gene expression is not because of a general upregulation of the inflammatory response. This analysis suggests that H3K9me2 acts to specifically repress the induction of target genes in response to inflammatory signaling.

To assess whether MMP regulation by H3K9me2 could affect VSMC extracellular matrix remodeling, we examined whether G9A/GLP inhibition affects matrix degradation. We treated cultured murine VSMCs with IL-1α and UNC before incubating with dye-quenched gelatin, which is converted into fluorescent peptides upon digestion. Inhibition of G9A/GLP further increased IL-1α-induced gelatin digestion (1.6-fold) compared with VSMCs treated with IL-1α alone (Figure 3D), indicating that H3K9me2-regulation of MMP expression in VSMCs might affect vascular remodeling in disease.

### VSMC-Specific Upregulation of Inflammation-Responsive H3K9me2-Target Genes After Carotid Ligation Is Enhanced by G9A/GLP Inhibition

**Figure 4.**
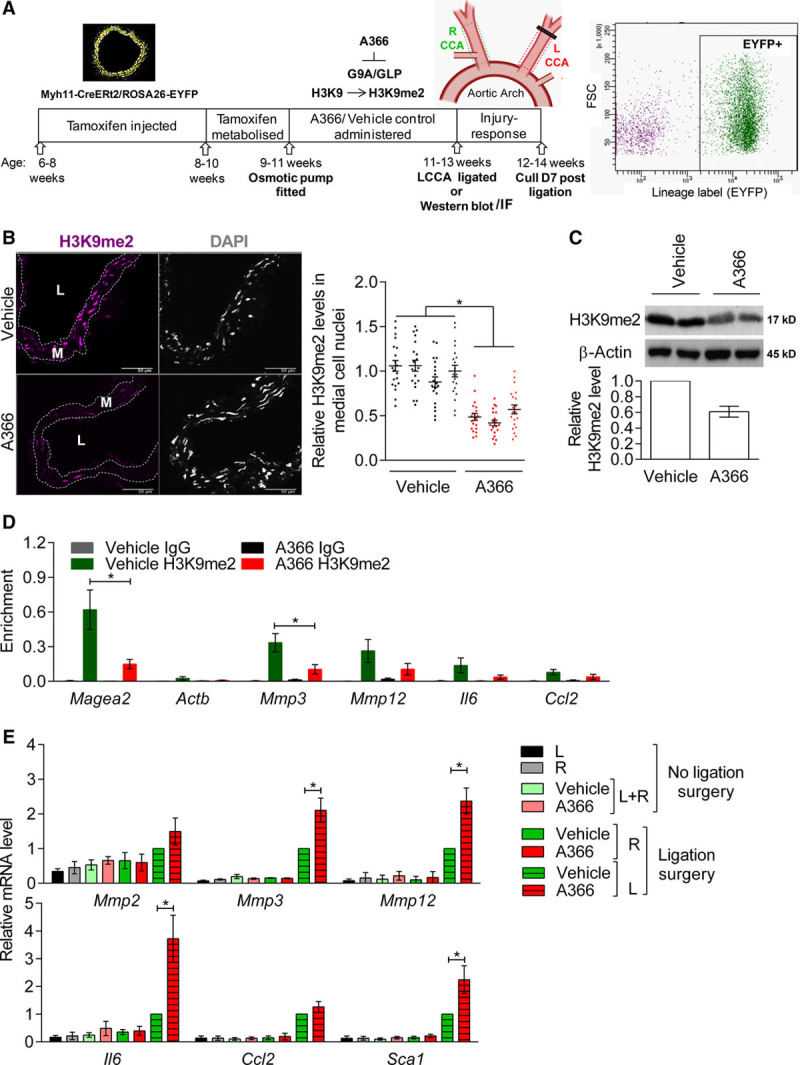
**Vascular smooth muscle cell (VSMC)-specific upregulation of histone H3 lysine 9 dimethylation (H3K9me2)-target genes after carotid ligation is enhanced by G9A/GLP (G9A-like protein) inhibition.** **A**, Schematic of experimental model. Lineage-labeled *Myh11-Cre*^*ERt2*^*/ROSA26-EYFP* (enhanced yellow fluorescent protein) mice were administered A366 (30 mg/kg per d) or vehicle control and subjected to ligation of the left common carotid artery (CCA). **B**, Representative immunofluorescence staining and quantification of H3K9me2 in left (L) CCA cryosections from mice administered vehicle control or A366 for 14 d. Signals for H3K9me2 (magenta) and DAPI (4′,6-diamidino-2-phenylindole; white) are shown. Scale bars, 50 μm. Dot plot shows H3K9me2 signal in individual medial cell nuclei, defined by their location between elastic lamina, relative to the average H3K9me2 signal in vehicle controls analyzed in the same batch (batches are indicated with symbols). Mean (line) and SEM (error bars) are indicated. n=4 animals for vehicle, n=3 animals for A366. *P<0.05 (linear model, see online-only Data Supplement). **C**, Representative western blot and quantification of H3K9me2 levels in aortic medial VSMCs of mice administered vehicle control or A366 for 14 days. Data (mean±SEM of 3 biological replicates) are shown relative to vehicle control, normalized to β-actin. **D**, Chromatin immunoprecipitation-qPCR analysis of the aortic medial layer (adventitial and endothelial cells removed) from mice 21 days after insertion of an osmotic pump delivering A366 or vehicle control. H3K9me2 enrichment at control loci (*Magea2*, positive and *Actb*, negative) and *Mmp3*, *Mmp12*, *Il6*, and *Ccl2* promoters is shown alongside negative control IgG. Mean±SEM of 5 to 6 biological replicates. *P<0.05 (Kruskal-Wallis). **E**, RT-qPCR (reverse transcription with quantitative polymerase chain reaction) analysis of *Mmp2*, *Mmp3*, *Mmp12*, *Il6, Ccl2*, and *Sca1 (Ly6a*) in EYFP^+^ carotid VSMCs isolated by flow cytometry 7 d after carotid ligation. *Mmp9* transcripts were not detected in any sample. Data (mean±SEM of 4 experiments) are shown relative to ligated LCCA from vehicle control (striped, green bars) and normalized to housekeeping genes (*Hprt1* and *Hmbs*). *P<0.05 (Kruskal-Wallis). L indicates left, and R, right CCA.

We next assessed the effect of reducing H3K9me2 in vivo on injury-induced MMP expression following carotid artery ligation (Figure 4A). VSMC-lineage-labeled animals (*Myh11-Cre*^*ERt2*^*/Rosa26-EYFP*) were administered A366, a G9A/GLP inhibitor that displays similar specificity and efficacy but improved pharmacokinetics compared with UNC0638^43, 44^. Two weeks of A366 treatment resulted in reproducible reduction of global H3K9me2 levels in VSMCs as revealed by immunostaining (45%, Figure 4B) and Western blotting (40%, Figure 4C), compared with vehicle-treated controls. Subsequent to A366 administration, the left common carotid artery was ligated and vessels were analyzed 7 days later (Figure 4A). We confirmed by ChIP that H3K9me2 levels were reduced at target gene promoters at the end of the protocol (Figure 4D). Interestingly, analysis of control animals revealed that H3K9me2 was also enriched at the *Il6* promoter in VSMCs within tissue (Figure 4D, green bars), contrasting with observations in cultured cells (Figure 3B).

To assess the specific effect of H3K9me2 on injury-induced gene regulation in VSMCs, we performed reverse transcription with quantitative polymerase chain reaction on lineage-labeled, EYFP^+^ VSMCs isolated by flow cytometry from ligated and control carotid arteries 7 days after surgery. Compared with vehicle-treated control mice, injury-induced expression of *Mmp3*, *Mmp12*, and *Il6* was significantly increased in those treated with A366 (Figure 4E), correlating with reduced H3K9me2 at their promoters (Figure 4D). Interestingly, A366 also significantly increased expression of *Sca1/Ly6a*, a marker of phenotypically switched VSMCs,^35^ suggesting an increased VSMC response to injury compared with controls (Figure 4E). We confirmed that flow cytometry-purified VSMC cell populations did not express markers of adventitial fibroblasts (*Pdgfra*), endothelial cells (*Cd31*), or bone marrow-derived cells (*Cd45*), beyond background (Figure III in the online-only Data Supplement). Importantly, A366 did not affect expression of H3K9me2 target genes in uninjured vessels, the response of *Ccl2* to ligation-injury, or the reduction of contractile VSMC gene expression after ligation (Figure 4E; Figure III in the online-only Data Supplement). Together, these results suggest that H3K9me2 attenuates induction of inflammation-responsive H3K9me2 target genes in VSMCs in vivo and inhibits VSMC phenotypic switching to a more proinflammatory state.

### H3K9me2-Mediated Regulation of Inflammation-Associated VSMC Gene Expression Changes Is Conserved in Humans

**Figure 5.**
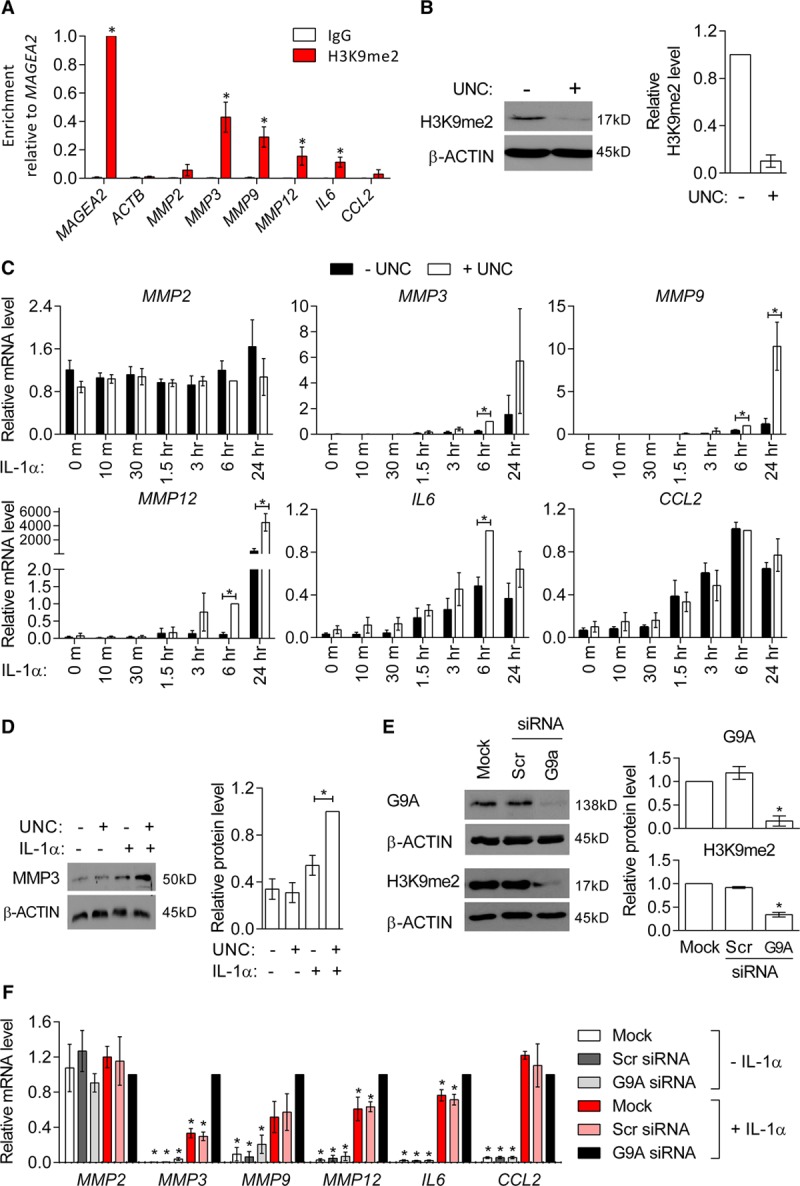
**Histone H3 lysine 9 dimethylation (H3K9me2)-mediated regulation of inflammation-associated genes is conserved in human aortic VSMCs (hVSMCs).** **A**, Chromatin immunoprecipitation-qPCR analysis in cultured hVSMCs, showing enrichment for H3K9me2 relative to *MAGEA2* (positive control) at *ACTB* (negative control) and *MMP2*, *MMP3*, *MMP9*, *IL6*, and *CCL2* promoters compared with signals for negative control IgG (immunoglobulin). Graph show mean±SEM of 6 experiments. *P<0.05 (Kruskal-Wallis, H3K9me2 enrichment compared with *ACTB*). **B**, Representative western blot and densitometric analysis of H3K9me2 levels in control (−UNC [UNC0638]) and UNC-treated (+UNC) cultured hVSMCs. Data (mean±SEM of 3 experiments) are shown relative to untreated cells and normalized to β-actin. **C**, RT-qPCR (reverse transcription with quantitative polymerase chain reaction) analysis of *MMP2*, *MMP3*, *MMP9*, *MMP12*, *IL6*, and *CCL2* in control hVSMCs and after IL-1α treatment for 10, 30 min, 1.5, 3, 6, or 24 h, without (−UNC, black bars) and with prior UNC treatment (+UNC, white bars). Data are relative to cells treated with UNC+IL-1α for 6 h and normalized to housekeeping genes (*HMBS* and *YWHAZ*). Graph show mean±SEM of 4 to 10 experiments. *P<0.05 (Kruskal-Wallis). **D**, Representative western blot and densitomeric analysis of MMP3 in control, UNC, IL-1α, and UNC+IL-1α-treated cultured hVSMCs. Mean±SEM of 3 experiments is shown relative to cells treated with UNC+IL-1α and normalized to β-actin. *P<0.05 (Kruskal-Wallis). **E**, Representative western blot and densitometric analysis of G9A and H3K9me2 levels in mock, scrambled (Scr) siRNA, and G9A siRNA transfected hVSMCs. Data are shown relative to mock-transfected hVSMCs and normalized to β-actin (mean±SEM of 4 experiments). *P<0.05 (Kruskal-Wallis, compared with Scr). **F**, RT-qPCR analysis of *MMP2*, *MMP3*, *MMP9*, *MMP12*, *IL6*, and *CCL2* in mock, Scr siRNA and G9A siRNA-transfected hVSMCs with and without IL-1α stimulation. Data (mean±SEM of 4 experiments) are relative to hVSMCs treated with G9A siRNA+IL-1α and normalized to housekeeping genes (*HPRT1* and *YWHAZ*). *P<0.05 (Kruskal-Wallis, compared with G9A siRNA+IL-1α). Each experiment used hVSMC isolates derived from different individuals.

ChIP analysis in primary hVSMC cultures revealed that enrichment of H3K9me2 at the *MMP3*, *MMP9*, and *MMP12* gene promoters compared with the active *ACTB* control locus with and without IL-1α treatment was conserved in human (Figure 5A; Figure IV in the online-only Data Supplement). Similar to murine tissue VSMCs, *IL6* was also a H3K9me2 target in hVSMCs, whereas this repressive epigenetic mark was not present at *CCL2* and *MMP2* promoters (Figure 5A). UNC treatment reduced global H3K9me2 levels 10-fold (Figure 5B) and potentiated the IL-1α response of H3K9me2 target genes at both the transcript and protein level (Figure 5C, D). This was most pronounced 6 hours after IL-1α stimulation for *IL6*, when expression peaked, whereas potentiation of *MMP3*, *MMP9*, and *MMP12* transcript levels by UNC was also observed 24 hours after stimulation (Figure 5C).

To ensure that the observed changes in gene expression were because of specific effects of UNC, we used siRNA to silence the G9A subunit of the heterodimeric G9A/GLP H3K9-methyltransferase complex. G9A knockdown resulted in > 7.5-fold reduction in G9A protein levels, which lead to a 2.7-fold reduction in global H3K9me2 levels compared with scrambled siRNA-transfected cells (Figure 5E). G9A knockdown alone did not influence basal transcript levels but enhanced IL-1α-induced *MMP3, MMP9, MMP12* and *IL6* expression (Figure 5F). Consistent with the effect of UNC, siRNA-mediated G9A knockdown did not affect the expression of *MMP2* or the response of *CCL2* to IL-1α (Figure 5F).

### H3K9me2 Does Not Affect Inflammation-Induced Signal Transduction

In addition to their role as histone methyltransferases, G9A/GLP have nonhistone targets,^45^ which may influence protein stability,^46^ protein-protein interactions,^47,48^ and cellular signaling pathways.^45,49^ We therefore tested whether reducing G9A/GLP activity affects signaling cascades downstream of IL-1α (Figure VA in the online-only Data Supplement). Expression levels of the IL-1 receptor was modestly increased in IL-1α-treated hVSMCs but was not affected by UNC-mediated G9A/GLP inhibition (Figure VB in the online-only Data Supplement). Similar to IL-1α, TNF-α-mediated induction of H3K9me2 target genes *MMP3*, *MMP9*, *MMP12*, and *IL6* was also potentiated by UNC, whereas *MMP2* or *CCL2* expression was not affected (Figure VC in the online-only Data Supplement). This suggests that UNC acts downstream of receptor binding and affects a factor that is common to TNF-α and IL-1α signaling such as NFκB and MAPK-mediated activation of the AP-1 transcription factor (Figure VA in the online-only Data Supplement). Binding of NFκB and AP-1 transcription factors to a number of MMP and proinflammatory cytokine promoters, including *MMP3*^50-52^, *MMP9*^51^, *MMP12*,^51^ and *IL6*^53^, has been reported as necessary to induce their expression.

To investigate whether G9A/GLP regulates inflammation-induced NFκB activity, we analyzed regulation of the p65 subunit, which contains the transactivation domain necessary for gene induction.^54^ Compared with untreated control cells, p65 levels (Figure VI A in the online-only Data Supplement) were unchanged in hVSMCs treated with UNC±IL-1α. UNC treatment neither affected the nuclear-to-cytoplasmic ratio of p65 at basal conditions nor its nuclear translocation in response to IL-1α or TNF-α stimulation (Figure VI in the online-only Data Supplement). These results suggest that potentiation of IL-1α/TNF-α-induced *IL6* and *MMP* expression by UNC is not because of increased upstream NFκB signaling.

Inflammatory cytokines induce phosphorylation of MAPKs at specific residues, which ultimately leads to phosphorylation of AP-1 subunits, such as cJUN.^55–57^ Western blotting showed that neither timing nor levels of phosphorylated JNK (cJUN N-terminal kinase), ERK (extracellular regulating kinase) 1/2, and p38 kinase (p38) were affected by UNC treatment before IL-1α stimulation (Figure VII in the online-only Data Supplement), suggesting that G9A/GLP inhibition does not influence MAPK activation.

### H3K9me2 Attenuates NFκB and AP-1 Binding to Inflammation-Responsive Target Gene Promoters

**Figure 6.**
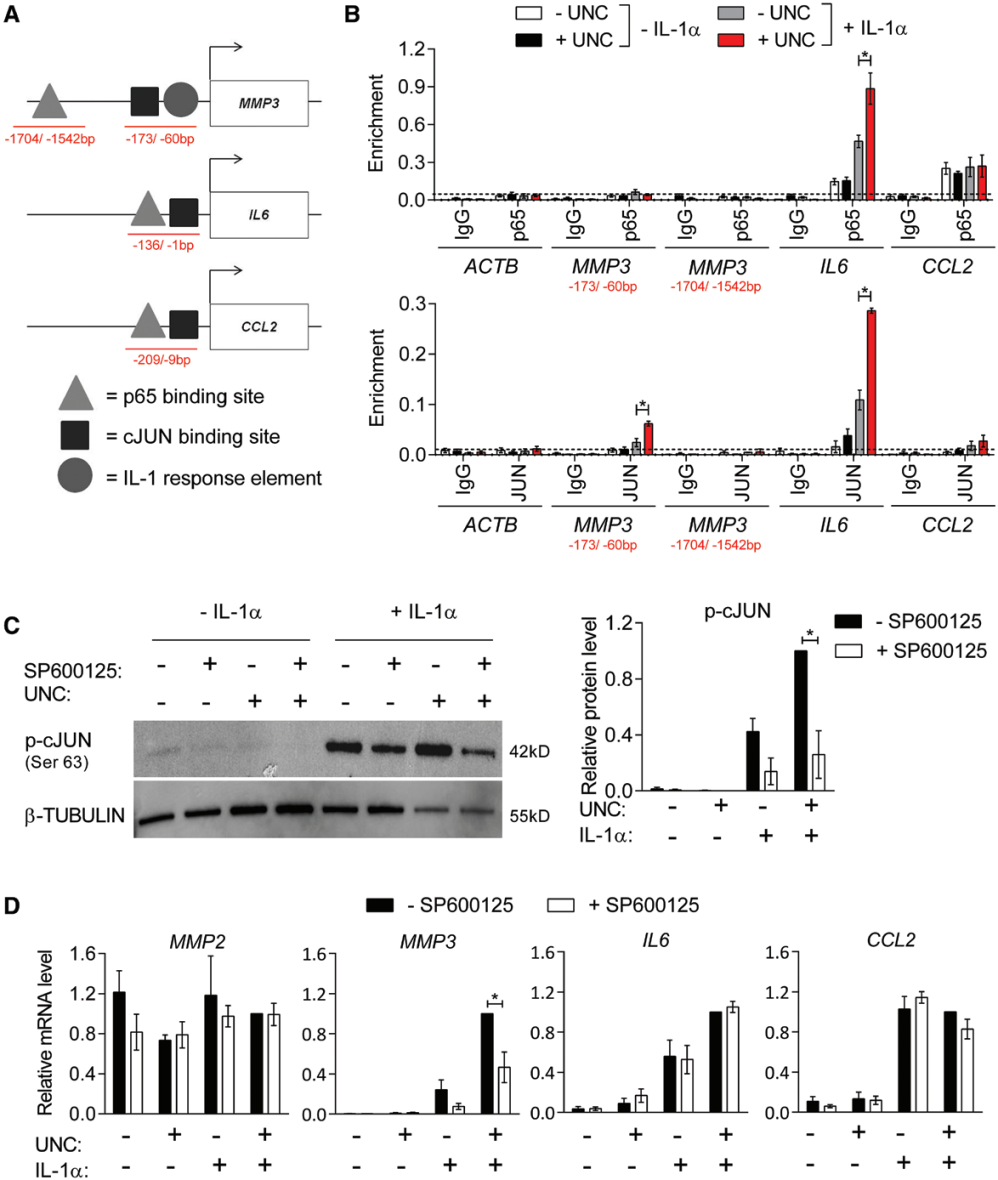
**IL (interleukin)-1α-induced binding of p65 and cJUN at histone H3 lysine 9 dimethylation (H3K9me2)-target genes is enhanced by G9A/GLP (G9A-like protein) inhibition.** **A**, Schematic indicating positions of reported p65 and cJUN binding sites and an IL-1-responsive element at the *MMP3* (Borghaei et al, Clark et al, Quinones et al),^41–43^ *IL6* (Gomard et al),^44^ and *CCL2* (Sutcliffe et al)^49^ promoters. Red lines indicate chromatin immunoprecipitation (ChIP)-qPCR amplicons. **B**, ChIP-qPCR analysis of p65 and cJUN binding relative to input in untreated control (white bars), UNC (UNC0638; black), IL-1α (gray), and UNC+IL-1α-treated human aortic VSMCs (hVSMCs; red) showing enrichment levels at the reported binding sites within the *MMP3*, *IL6*, and *CCL2* promoters and the *ACTB* negative control locus compared with negative control IgG (immunoglobulin). Background levels of p65 binding, indicated by a line, are based on enrichment at *ACTB*. Graph shows mean±SEM of 4 experiments. **C**, Representative western blot and densitometric analysis p-cJUN (Ser63) in untreated control hVSMCs and cells stimulated with IL-1α, without and with prior treatment with UNC and SP600125 for 48 h. Data (mean±SEM of 4 experiments) are relative to +UNC+IL-1α-SP600125 and normalized to β-TUBULIN levels. **D**, RT-qPCR (reverse transcription with quantitative polymerase chain reaction) analysis of *MMP2*, *MMP3*, *IL6*, and *CCL2* in control and IL-1α-treated hVSMCs, without and with prior UNC and/or SP600125 treatment. Data (mean±SEM of 4 experiments) are relative to +UNC+IL-1α−SP600125 and normalized to the average of 2 housekeeping genes (*HPRT1* and *YWHAZ*). Each experiment used hVSMCs derived from different individuals; *P<0.05 (Kruskal-Wallis).

Binding sites for NFκB and AP-1 and IL-1-responsive elements have been reported within 2 kb of the transcriptional start site of *MMP3*, *IL6, and CCL2* (Figure 6A).^50–53,58^ To investigate whether H3K9me2 affects transcription factor binding at target gene promoters, we performed ChIP for p65 (NFκB) and cJUN (AP-1) in hVSMCs treated with IL-1α and UNC. Compared with the *ACTB* negative control locus, p65 was enriched at the *IL6* and *CCL2* gene promoters in untreated cells. At the *IL6* locus, p65 binding was increased by IL-1α stimulation, and this was significantly enhanced by UNC pretreatment (Figure 6B). Increased p65 binding in UNC-treated cells correlated with reduced H3K9me2, suggesting that H3K9me2 at the *IL6* locus prevents NFκB binding to its cognate sequence. In contrast, the enrichment of p65 at *CCL2*, which is not a H3K9me2 target (Figure 5A), was unaffected by inhibition of G9A/GLP (Figure 6B).

Notably, p65 enrichment was not detected at the reported IL-1-responsive element in the proximal *MMP3* promoter,^51,52^ nor at a reported NFκB site 1.5 kb upstream of the transcriptional start site,^50^ irrespective of cell stimulation (Figure 6B). In contrast, binding of the AP-1 subunit cJUN was detected at both *IL6* and *MMP3* promoters on IL-1α stimulation, and cJUN enrichment was significantly increased by UNC-mediated H3K9me2 depletion (Figure 6B). No cJUN binding was observed at the *CCL2* promoter under any condition (Figure 6B).

To explore the impact of differences in transcription factor binding at the *MMP3* and *IL6* loci, we measured the sensitivity of IL-1α-induced transcription to inhibition of JNK (SP600125^59^), the primary MAPK responsible for cJUN phosphorylation at Ser63.^60^ Treatment with SP600125 significantly reduced IL-1α-induced p-cJUN levels (3.9-fold to 4.7-fold; Figure 6C). Interestingly, G9A/GLP inhibition caused a modest (2-fold) increase in p-cJUN in hVSMCs stimulated with IL-1α, despite not affecting the levels of phosphorylated MAPKs (Figure 6C; Figure VII in the online-only Data Supplement) or total cJUN (Figure VII in the online-only Data Supplement). Reduced levels of p-cJUN in SP600125-treated cells selectively affected *MMP3* gene expression resulting in an attenuated induction by IL-1α, whereas IL-1α-induced *IL6* and *CCL2* expression was not affected by JNK inhibition (Figure 6D). These results suggest that JNK-mediated cJUN phosphorylation is necessary for IL-1α-induced expression of *MMP3* but not required for activation of the NFκB target *IL6*.

Taken together, these findings suggest that H3K9me2 acts directly at the *MMP3* and *IL6* promoter to restrain IL-1α-induced upregulation by inhibiting NFκB (p65) and AP-1 (cJUN) transcription factor binding and demonstrates that inflammation-induced expression of *MMP3*, *IL6*, and *CCL2* are regulated via different mechanisms.

## Discussion

We here demonstrate an evolutionary conserved role of H3K9me2 in regulating the proinflammatory VSMC phenotype associated with vascular disease and identify a subset of inflammation-responsive genes, including *MMP3*, *MMP9*, *MMP12*, and *IL6*, as direct H3K9me2 targets in VSMCs. We show that loss of H3K9me2 increases binding of inflammation-induced transcription factors and increases expression of these *MMP* and proinflammatory VSMC genes in response to inflammatory stimuli both in vitro and in vivo. This functional role of H3K9me2 suggests that this epigenetic mark acts to restrain inflammation-induced, CVD-associated gene expression in VSMCs. It is therefore tempting to speculate that the reduced H3K9me2 levels we observed in atherosclerosis and after injury is required to elicit VSMC inflammatory activation and vascular remodeling. Increasing H3K9me2 levels by stimulating G9A/GLP activity or inhibiting H3K9me2-specific demethylases (eg, KDM3A) might therefore be a strategy for therapeutic targeting in CVD.

The global loss of H3K9me2 in VSMCs from atherosclerotic lesions and in arteries undergoing remodeling after vascular injury is consistent with the reported H3K9me2 reduction in atherosclerotic plaques from patients compared with healthy human arteries.^22^ Our analysis of lineage traced VSMCs supports the observation that αSMA-positive plaque cells have lower H3K9me2 levels^61^ and further indicates that this epigenetic mark is also reduced in VSMC-derived cells within the lesion core, that do not express contractile markers.

H3K9me2-mediated regulation of inflammation-induced genes in VSMCs might also explain the accelerated neointima formation following vascular injury previously detected in diabetic rats when H3K9me2 levels were reduced by overexpression of KDM3A, a H3K9me2-specific demethylase.^62,63^ VSMCs from diabetic patients also display reduced levels of H3K9me2 compared with nondiabetic controls, and KDM3A is induced by high insulin levels.^62,64^ Therefore, dysregulation of H3K9me2 might contribute to diabetes-associated vascular complications.

An inverse correlation between H3K9me2 occupancy at *MMP* and other inflammatory gene promoters and their expression has also been reported in other cell types.^24–27^ For example, H3K9me2 depletion at the *Mmp9* promoter correlates with elevated *Mmp9* expression in retinal endothelial cells from diabetic rats compared with controls.^27^ Similarly, solar-stimulated UV radiation induces loss of H3K9me2 at the *MMP3* promoter in primary human dermal fibroblasts, correlating with enhanced *MMP3* mRNA levels.^25^ In addition, genetic ablation or pharmacological inhibition of G9A/GLP stimulates the expression of inflammatory genes in mouse embryonic fibroblasts.^24^ Similar to our findings in VSMCs, reduction of H3K9me2 levels in fibroblasts potentiates poly(I:C)-induced upregulation of inflammatory genes.^24^ Moreover, G9A-deficient flies are hypersensitive to RNA virus infection and succumb faster to infection than wild-type controls.^28^ These observations suggest that G9A/GLP represents an evolutionarily conserved mechanism to control genes involved in processes that require tight and dynamic regulation—such as the inflammatory response—in a variety of cell types.

We demonstrate increased binding of AP-1 and NFκB specifically at H3K9me2 target genes on reduction of H3K9me2 levels following G9A/GLP inhibition (see Graphical Abstract). In contrast, inhibition of G9A/GLP did not affect IL-1α/TNF-α-induced activation of upstream signaling, including p65 activation and phosphorylation of p38, JNK, and ERK1/2. MAPKs can phosphorylate substrates once bound to chromatin.^65^ We propose that the 2-fold increase in p-cJUN in IL-1α-stimulated cells after G9A/GLP inhibition could be because of increased phosphorylation efficiency of chromatin-associated cJUN, which potentiates transactivation.^66,67^ Alternatively, G9A/GLP might regulate an as-yet-unidentified cJUN phosphatase.^66^ Regardless, our data demonstrate that key inflammation-induced genes have distinct regulatory mechanisms in VSMCs. Non-H3K9me2 targets such as *CCL2* display transcription factor binding at basal levels resulting in rapid induction upon stimulation. In contrast, H3K9me2 target gene induction is delayed in a context-specific manner, which could depend on both specific transcription factor usage and locus-specific chromatin regulation.

In conclusion, our data implicate H3K9me2 as a functional epigenetic regulator of the VSMC inflammatory response. We propose that H3K9me2 acts to prevent spurious induction of CVD-associated proinflammatory genes, including *MMPs* and *IL6*. Differential transcriptional regulation of selective *MMPs* and other inflammation-induced genes could be leveraged for developing more specific drugs for future clinical use.

## Acknowledgments

We thank the Cambridge Statistics Clinic (Department of Statistics) for advise on statistical analysis, Mikhail Spivakov for advise on chromatin immunoprecipitation-seq data analysis, Gregory Strachan and the Wellcome Trust-Medical Research Council, Institute of Metabolic Science, Metabolic Research Laboratories, Imaging core (Wellcome Trust Major Award [208363/Z/17/Z]) for technical assistance, the Cambridge National Institute for Health Research Biomedical Research Centre Cell Phenotyping Hub for cell sorting and all members of the Jørgensen group for helpful discussions.

## Sources of Funding

J.L. Harman was funded by a British Heart Foundation (BHF) studentship (FS/15/38/31516); L. Dobnikar was funded by a BBSRC DTP studentship; J. Chappell was funded by the BHF Centre for Cardiovascular Research Excellence (RE/13/6/30180); B.G. Stokell was funded by a studentship from the Cantab Capital Institute for the Mathematics of Information. K. Foote was funded by the BHF PG/16/63/32307; A. Finigan and A. Uryga were funded by the BHF RG/13/14/30314. A.L. Taylor was funded by BHF studentship (FS/15/62/32032). M.R. Bennett and H.F. Jørgensen were supported by the BHF (CH/2000003), the Oxbridge BHF Centre for Regenerative Medicine (RM/13/3/30159, RM/17/2/33380), the BHF Centre for Cardiovascular Research Excellence (RE/13/6/30180), and the National Institute for Health Research Cambridge Biomedical Research Center.

## Disclosures

None.

## Supplementary Material
