# Supplementary material for: Epigenetic Regulation of Vascular Smooth Muscle Cells by Histone H3 Lysine 9 Dimethylation Attenuates Target Gene-Induction by Inflammatory Signaling
Source: Arterioscler Thromb Vasc Biol. 2019 Aug 22;39(11):2289–302. doi: 10.1161/ATVBAHA.119.312765 (PMC6818986; doi:10.1161/ATVBAHA.119.312765)
Supplement: Supplementary file 1 [file atv-39-2289-s001.pdf]

Supplemental Material

Epigenetic Regulation of Vascular Smooth Muscle Cells by Histone H3 Lysine 9 Dimethylation Attenuates Target Gene-Induction by Inflammatory Signaling

Jennifer L. Harman[1], Lina Dobnikar[1,2], Joel Chappell[1], Benjamin G. Stokell[3], Amanda Dalby[1,†], Kirsty Foote[1], Alison Finigan[1], Paula Freire-Pritchett[2,‡], Annabel L. Taylor[1], Matthew D. Worssam[1], Ralitsa R. Madsen[1,#], Elena Loche[1], Anna Uryga[1], Martin R. Bennett[1], Helle F. Jørgensen[1, *]

[1]Division of Cardiovascular Medicine, Department of Medicine, University of Cambridge, UK.
[2]Babraham Institute, Babraham Research Campus, Cambridge, UK.
[3]Statistical Laboratory, Centre for Mathematical Sciences, University of Cambridge, UK

[†]Current address: Institute of Cardiovascular Sciences, College of Medical and Dental Sciences, University of Birmingham, UK.
[‡]Current address: Cell Biology Division, MRC Laboratory of Molecular Biology, Cambridge, UK.
[#]Current address: Centre for Cardiovascular Science, Queen's Medical Research Institute, University of Edinburgh, UK

Supplemental Methods

Supplemental Tables I-VIII

I: H3K9me2-enriched arteriosclerosis-associated gene promoters
II: Number of CCAs pooled per *in vivo* RT-qPCR experiment
III: Primary antibodies
IV: Secondary antibodies
V: Mouse ChIP-qPCR primer sequences
VI: Human ChIP-qPCR primer sequences
VII: Mouse RT-qPCR primer sequences
VIII: Human RT-qPCR primer sequences

Supplemental Figures I-V

I: Global levels of H3K9me2 are reduced in VSMCs upon vascular injury
II: ChIP-seq for H3K9me2 in ex vivo murine VSMCs
III: VSMC-specific upregulation of H3K9me2-target genes after carotid ligation is enhanced by G9A/GLP inhibition
IV: H3K9me2 is enriched at a subset of IL-1α responsive gene promoters in human VSMCs
V: The effect of G9A/GLP inhibition is shared by IL-1α and TNF-α
VI: G9A/GLP inhibition does not affect NFκB signalling in cultured hVSMCs
VII: G9A/GLP inhibition does not affect cJun signalling in cultured hVSMCs

Supplemental References

Supplemental methods

Animal experiments

All experimental animals were males as the *Myh11-Cre*^{ERt2} transgene is Y-linked. VSMC lineage labelling of *Myh11-Cre*^{ERt2}+ animals was achieved by intraperitoneal tamoxifen injections (10 injections of 1 mg each) and animals rested for at least one week before further manipulation to allow tamoxifen washout. *Myh11-Cre*^{ERt2}*/Rosa26-Confetti*$^{+}$*/Apoe*$^{-/-}$ animals were fed a standard chow or an atherosclerosis-inducing diet (Special Diet Services, 829100, Western Rd pellets; 21% fat and 0.2% cholesterol) subsequent to tamoxifen treatment (3-4 months of high fat diet feeding for animals analyzed by immunostaining and 6 months for animals analyzed by western blotting). For carotid ligation experiments, mice were given a pre-operative injection of buprenorphine (0.1 mg/kg; subcutaneously) and anaesthetized with 2.5% inhalable isofluorane (maintained at 1.5%). The left common carotid artery was exposed and ligated just below the bifurcation with a 6-0 silk suture. The left common carotid artery was tied firmly with one knot using 6-0 silk suture just below the bifurcation point. Both the right and left carotid arteries were removed at the indicated time post-surgery and processed for microscopy or flow cytometry assisted cell sorting (FACS) of lineage labelled VSMCs. For *in vivo* administration of the G9A/GLP inhibitor A366, mini osmotic pumps (2002, Azlet) filled with 30 mg/kg/day A366 dissolved in 98:2 PEG 400/polysorbate 80 or vehicle only (98:2 PEG 400/polysorbate 80) were implanted subcutaneously into animals anaesthetized with 2.5% inhalable isofluorane (maintained at 1.5%) with pre-operative administration of Buprenorphine (0.1 mg/kg; subcutaneously). The dose of A366 is identical to what was previously used to reduce H3K9me2 levels in cardiomyocytes[1]. We did not observe any difference in weight gain between A366 and vehicle-treated animals (Supplemental Figure III C). Where possible, the researcher was blinded to treatment group (A366 versus vehicle control). In some animals, the aorta and right and left common carotid arteries were removed 14 days post-surgery and processed for western blot or immunofluorescence to analyze global H3K9me2 levels in the VSMCs. Alternatively, animals were subjected to carotid ligation 14 days after osmotic pump insertion as described above and arteries were analyzed 7 days post ligation. For each experiment a pool of 2-3 CCAs were used as detailed in Supplemental Table II.

Chromatin Immunoprecipitation (ChIP) for histone modifications

Cultured VSMCs and *ex vivo* tissue from the medial layer of the aorta were washed in phosphate buffered saline (PBS) and fixed in 1% formaldehyde in PBS for 10 minutes at room temperature before quenching with 0.125 mM glycine for 5 minutes. Subsequently, the cells/tissue were washed three times in PBS. *Ex vivo* tissue was snap frozen and homogenised using a metal mortar. The cells/homogenised tissue were resuspended in nuclear lysis buffer (50 mM Tris-HCl pH 8.1, 10 mM EDTA pH 8.0, 1% SDS) supplemented with protease inhibitors (Roche) and incubated on ice for a minimum of 5 minutes. A Bioruptor Pico sonication device (Diagenode) set at high power, 30 seconds on and 30 seconds off for 15 to 20 cycles was used to fragment the chromatin to approximately 0.2-1 kb. The sonicated chromatin was centrifuged at 12,000 g at 4 ºC for 15 minutes to remove insoluble debris. The DNA concentration was determined using a Nanodrop ND-1000 spectrometer. Fragmentation of chromatin was confirmed by Bioanalyzer assessment (DNA High Sensitivity Analysis Kit Agilent Technologies) of a 0.5 µg aliquot after de-crosslinking. To de-cross link, the 0.5 µg aliquots were incubated in elution buffer (20 mM Tris-HCl pH8.0, 5 mM EDTA pH 8.0, 50 mM NaCl, 1% SDS, 50 µg/ml Proteinase K, 100 µg/ml RNase A) at 37 ºC for an hour and then at 68 ºC overnight. Sonicated chromatin (2-20 µg) was incubated with the appropriate antibodies (Table III) or control IgG (Table IV) with BSA (100 µg/ml) and yeast tRNA (20 µg/ml), diluted 10x in RIPA-140 (10 mM Tris-HCl pH 7.5, 1 mM EDTA pH 8.0, 0.5 mM EGTA, 1% Triton X-100, 0.10% SDS, 0.10% sodium deoxycholate, 140 mM NaCl) supplemented with protease inhibitors (Roche), overnight at 4 ºC. A 10% aliquot of the chromatin used for each ChIP was directly de-crosslinked as described above. Protein A and protein G magnetic beads (600 µg, Life Technologies), washed twice in RIPA-140 buffer were added to the samples and incubated at 4 ºC on a rotating wheel for 2 hours. Samples were then washed twice in RIPA-140, twice in RIPA-500 (10 mM Tris-HCl pH 7.5, 1 mM EDTA pH 8.0, 0.5 mM EGTA, 1% Triton X-100, 0.10% SDS, 0.10% sodium deoxycholate, 500 mM NaCl), RIPA-LiCl (10 mM Tris-HCl pH 7.5, 1 mM EDTA pH 8.0, 0.5 mM EGTA, 1% Triton X-100, 0.10% SDS, 0.10% sodium deoxycholate, 250

mM LiCl) and twice in TE (10 mM Tris-HCl pH 8.1, 1 mM EDTA pH 8.0) before being resuspended in 200 µl of elution buffer. Samples were incubated in elution buffer at 37 ºC for an hour and then 68 ºC overnight. The gDNA samples were purified using a gel extraction kit (Qiagen) following the manufacturer's protocol and eluted in 50-100 µl of TE. The purified gDNA was analyzed by qPCR using primers listed in Tables V and VI.

ChIP for NFkB-p65 and AP1-cJun
Human VSMCs (3-4 million per ChIP) were washed in PBS and fixed with 1% formaldehyde and Cross-link gold (Diagenode) following the manufacturers protocol. Fixed cells were incubated at room temperature for 30 minutes, then quenched with 0.125 M glycine for 15 min at room temperature. The cells were then lysed using buffers from the iDeal ChIP-seq Kit for Transcription Factors (Diagenode), following the manufacturer's instructions. The chromatin was sheared using a Bioruptor Pico sonication device (Diagenode) set at high power, 30 seconds on and 30 seconds off for 20 cycles, to fragment the chromatin to approximately 0.2-1 kb. Fragmentation of chromatin was confirmed by Bioanalyzer assessment (DNA High Sensitivity Analysis Kit Agilent Technologies) of a 0.5 µg aliquot after de-crosslinking. To de-cross link, the 0.5 µg aliquots were incubated in elution buffer (20 mM Tris-HCl pH8.0, 5mM EDTA pH 8.0, 50 mM NaCl, 1% SDS, 50 µg/ml Proteinase K, 100 µg/ml RNase A) at 37 ºC for an hour and then at 68 ºC overnight. Chromatin immunoprecipitation was carried out using the iDeal ChIP-seq Kit for Transcription Factors using the appropriate antibodies or control IgG (Table III, IV), according to the manufacturers protocol. The gDNA samples were purified using iPure magnetic beads and eluted in 40ul of the elution buffer provided in the iDeal ChIP-seq Kit for Transcription Factors (Diagenode). The purified gDNA was analyzed by qRT-PCR using primers listed in Table VI.

Flow cytometry
Carotid arteries were removed from animals culled by CO_2 asphyxiation, dissected free from adipose and connective tissue and digested in 2.5 mg/ml of collagenase II (Invitrogen) and 2.5 U/ml of elastase (Worthington) in DMEM at 37 ºC, 5% CO_2 (0.5 ml per carotid artery) for approximately 1 to 3 hours, mixing every 30 minutes, until a single cell suspension was reached. The dissociated cells were washed once in PBS, resuspended in 200 µl of FACS buffer (1% BSA in PBS), filtered through a 40 µm cell strainer and $EYFP^+$ VSMC were isolated on an Aria-fusion flow cytometry-assisted cell sorter (BD Bioscience).

Tissue processing for immunostaining
Carotid arteries were removed from animals culled by CO_2 asphyxiation and dissected free from adipose and connective tissue, fixed with 4% (v/w) paraformaldehyde in PBS for 20 minutes at room temperature, washed for 5 minutes in PBS, incubated in 30% sucrose in PBS at 4 ºC overnight, embedded in TissueTek O.C.T. and snap frozen using liquid nitrogen. The arteries were cut transversely into 14 µm thick sections, mounted on SuperFrost Plus Adhesion microscope slides (Thermo Scientific) and stored at -80 ºC. Sections shown in Figure 1A are from the carotid arteries of animals fed a standard chow or atherosclerosis-incuding high fat diet.

Immunostaining of cryosections
Serial cryosections were rinsed in PBS, permeabilised in 0.5% Triton x-100 in PBS for 30 minutes at room temperature and blocked for 1 hour at room temperature in 1% BSA, 10% Normal Goat Serum (DAKO/Agilent), 0.1% Triton x-100 in PBS. Sections were then incubated with anti-H3K9me2 antibody conjugated to Alexa Fluor-647 (1:100, ab203851 Abcam) in blocking buffer overnight at 4 ºC. The sections were then washed twice for 5 minutes in PBS before staining the nuclei with 300 nM of DAPI in PBS for 10 minutes. The sections were then washed twice in PBS and mounted in RapiClear® 1.52 (SunJin Lab). Stained cryosections were imaged using confocal laser scanning microscopy (Leica SP5 or SP8) with laser lines and detectors set for maximal sensitivity without spectral overlap for DAPI (405 laser, 417-508 nm), CFP (458 laser, 454-502 nm), GFP (488 laser, 498-506 nm), YFP (514 laser, 525-560 nm), RFP (561 laser, 565-650 nm) and Alexa Fluor-647 (633 laser, 650-700 nm). Cryosections were imaged using a 20x oil objective, and data was acquired at an optical section resolution of 1024 x 1024. Image stacks (12 µm) were collected with 1 µm z-steps. Image J software was used for image processing and analysis. VSMCs were identified by expression of the fluorescent Myh11-lineage labeling reporter and their

nuclei defined by DAPI staining. The mean pixel intensity of H3K9me2 staining in the nuclei of VSMCs was used to calculate H3K9me2 levels. Nuclei of individual Confetti+ cells included in the analysis were chosen without displaying the Alexa Fluor-647-H3K9me2 signal to avoid bias. A total of 20 (Figure 1A, 4A) or 30 (Figure 1C, Supplemental Figure 1B) Confetti+ VSMCs were analysed per animal (10 cells in each 12 μM image stacks per section, 2-3 sections per animal). Nuclear H3K9me2 levels for each individual VSMC was divided by the average VSMC nuclear H3K9me2 level of a control animal (standard diet, SD; no surgery left carotid artery or internal control right carotid artery) analysed in parallel.

Mouse VSMC Isolation
Whole aortas from C57BL/6J mice were dissected in PBS under an inverted Leica M80 microscope, applying aseptic technique. Fat and connective tissue were removed before the vessels were opened longitudinally to scrape off blood and endothelial cells. For primary mouse VSMC culture, each aorta was incubated in 1 ml of 1 mg/ml of collagenase II and 1 U/ml of elastase (Worthington) in DMEM at 37 ºC, 5% CO_2 for 10 minutes to peel off and remove the adventitia. Aortas for RNA extraction were stored in RNAlater (ThermoFisher, AM7024) at -20 ºC. For RNA extraction or ChIP, the adventitia was removed manually in PBS without enzymatic digestion. The isolated medial layer of VSMCs was then washed in PBS before being snap frozen in liquid nitrogen or being further processed for culture.

Primary mouse VSMC Culture
After isolating the medial layer of VSMCs, the tissue was further digested in 1 ml of 2.5 mg/ml of collagenase II (Invitrogen) and 2.5 U/ml of elastase (Worthington) in DMEM at 37 ºC, 5% CO_2 for approximately 1 to 3 hours, mixing every 20 minutes, until a single cell suspension was reached. The dissociated VSMCs were plated in VSMC growth medium (DMEM supplemented with 10% (v/v) of FBS, 100 U/ml of penicillin, 100 mg/ml of streptomycin and 2 mM of glutamine) into one well of a 12-well plate (Corning) per aorta. VSMCs were maintained at 37 ºC, 5% CO_2 in growth medium and split 1:2 every 3-5 days when approximately 90% confluent. For passaging, the cells were washed in PBS and incubated in trypsin at 37 ºC, 5% CO_2 for approximately 5 minutes. Experiments were carried out using passage 4 VSMCs.

Human VSMC Culture
Human aortic VSMCs were isolated from patients undergoing aortic valve replacement surgery with ethics committee approval. The gender and age of donors is provided in the Major Resources Tables. Adventitia and endothelial cells were manually removed from 2-3 mm² sections of aorta. The medial VSMC layer was then placed in a 6 well plate with a cover slip placed on top and cultured in growth medium (DMEM supplemented with 10% (v/v) of FBS, 100 U/ml of penicillin, 100 mg/ml of streptomycin and 2 mM of glutamine). VSMCs were cultured to approximately 90% confluence before passaging. For passaging, the cells were washed in PBS and incubated in trypsin at 37 ºC, 5% CO_2 for 3-5 minutes. Experiments were carried out using passage 6-15 VSMCs.

Treatment of VSMC cultures
Mouse and human VSMCs were seeded in 12-well plates in growth medium at a ratio of 1:2. When the cells became 70% confluent, the media was changed to chemically defined medium (CDM; 1:1 mixture of Iscove's modified Dulbecco's medium plus Ham's F-12 medium, both supplemented with GlutaMAX (Gibco, Life technologies), 5 mg/ml bovine serum albumin, 450 μM monothioglycerol, 15 mg/ml Transferrin, 7 mg/ml Insulin, 1 x lipids (100 x chemically defined lipid concentrate (Gibco, Life Technologies), 1 x penicillin/streptomycin (Gibco, Life Technologies). After 24 hours, the media was replaced with CDM with 1 μM UNC0638 (UNC, Tocris) and/or 10 μM SP600125 (Abcam) or vehicle control (DMSO) and the medium was refreshed a further 24 hours later. After 48 hours of treatment, 2 ng/ml of human recombinant IL-1α (Peprotech) or 90 ng/ml of human recombinant TNF-α (Peprotech) was added to the appropriate wells. After 6 hours of IL-1α or TNF-α stimulation, unless otherwise indicated in the figure legends, the cells were washed twice with PBS, harvested by trypsinisation, washed twice with PBS and cell pellets stored at -80 ºC.

G9A SiRNA knock down
Cultured human VSMCs (300,000 at passage 7-10) were plated per well of a 6 well plate (35 mm diameter) in VSMC growth medium. Eight hours after plating, the media was changed to CDM without penicillin/streptomycin and 16 hours later the cells were transfected with either a 100 nM pool of siRNA targeted against G9A (sc-43777, Santa Cruz) or a 100 nM pool of control scrambled siRNA (sc-37007, Santa Cruz) using HiPerfect transfection reagent (Qiagen), following the manufacturers protocol (12 µl of HiPerfect was used per transfection). 48 hours after transfection the cells were treated with or without 2 ng/ml of human recombinant IL-1α (Peprotech) for 6 hours, or as specified, before harvest.

Dye Quenched-gelatin Extra Cellular Matrix (ECM) degradation assay
Immediately after UNC0638 and/or IL-1α treatment of primary mouse VSMCs, 1 ml of 50 µg/ml of DQ-gelatin (Thermofisher Scientific), dissolved in CDM, was added per 9.5 cm^2 well. After 2 hours of DQ-gelatin treatment, the cells were washed twice in CDM before incubating with 300 nM of DAPI in PBS for 2 minutes. The cells were then washed once with PBS and mounted in VECTASHIELD Antifade mounting medium (Vector Laboratories). Degradation-induced fluorescence was used to measure MMP activity. The DQ-gelatin-treated VSMCs were imaged using a 20x objective with a fluorescence microscope (ZEISS Axio Vert A.1). The corrected total fluorescence (CTF) was quantified using ImageJ software as follows; CTF = [(area x mean pixel intensity) - (area x mean pixel intensity of 5 background readings)]/ cell number. Cell numbers were determined by counting DAPI stained nuclei. The average CTF of 5 fields of view was used to quantify the MMP activity of each sample.

Immunostaining of cultured VSMCs
After treatment of primary human VSMCs with UNC0638 (48h) and IL-1α or TNF-α (1h), the cells were washed twice with PBS and fixed with 4% paraformaldehyde for 10 minutes at room temperature. Cells were subsequently incubated with 0.5% Triton-X100 (Sigma) for 20 minutes at room temperature before being washed three times with PBS and blocked with 10% normal goat serum (Dako) in PBS for 1 hour at room temperature. Cells were then incubated with NFκB p65 antibody in blocking buffer (1 µg/ml, Santa Cruz sc-372) for 2 hours at room temperature and washed three times with blocking buffer. After 1 hour of incubation with Alexa Fluor 488-conjugated goat anti-rabbit IgG (2 µg/ml, Abcam ab150077) at room temperature, the cells were washed twice with PBS before incubating with 300 nM of DAPI in PBS for 2 minutes. Secondary only control stainings were done in parallel. Finally, the cells were then washed once with PBS and mounted in VECTASHIELD Antifade mounting medium (Vector laboratories). The cells were imaged using a 10x objective with a fluorescent microscope (ZEISS Axio Vert A.1). The nuclear/cytoplasmic ratio of p65 was measured using ImageJ software (http://dev.mri.cnrs.fr/projects/imagej-macros/wiki/Intensity_Ratio_Nuclei_Cytoplasm_Tool). The nuclear/cytoplasmic ratio measures the mean pixel intensity of nuclear area defined by DAPI divided by the mean grey value of the cytoplasmic area. The average nuclear/cytoplasmic p65 ratio of 3 fields of view was used to calculate the nuclear/cytoplasmic ratio of p65 of each sample.

Western blotting
Cell pellets were lysed directly in 2X Laemmli sample buffer (LSB, 20% glycerol, 4% SDS, 100 mM Tris HCl pH 6.8, 200 mM DTT), incubated at 98 ºC for 5 minutes and sonicated for 1 minute at medium intensity using a standard Bioruptor (Diagenode). For ex vivo tissue samples the adventitia was removed as described above. For the comparison of H3K9me2 levels in animals after HFD (Figure 1B) the tissue (medial layer, plaque, intima) were crushed using a pestle in 2 x LSB, incubated at 98 degrees and sonicated. For the analysis of A366-treated and control tissue (Figure 4C), the endothelial cell layer was manually removed by gentle scraping before processing. Total protein (10-30 µg) was resolved with SDS-PAGE and transferred to PVDF membranes. The membranes were incubated in blocking buffer (5% w/v nonfat dry milk in TBS (50 mM Tris-HCl, pH 7.5, 150 mM NaCl)) for one hour at room temperature. The membranes were then probed with primary antibodies (Table III) overnight at 4 ºC followed by secondary antibodies conjugated to horseradish peroxidase (HRP, Table IV) for 30-60 minutes at room temperature. All antibody incubation steps were done in blocking buffer. Protein was detected using the ECL system (GE Healthcare).

Reverse transcription - quantitative Real Time-Polymerase Chain Reaction (RT-qPCR)
Total RNA was extracted with TRIzol (Life Technologies) and 0.05-0.5 µg was reverse transcribed using a QuantiTect Reverse Transcription kit (Qiagen), according to the manufacturer's directions. For quantification, cDNA (diluted 1:2-1:10) was amplified, in technical duplicates, with a QuantiTect SYBR Green PCR kit (Qiagen) and primer pairs listed in Tables VII and VIII, using a Corbett Life Science Rotor Gene 6000 PCR system. A standard curve was used to calculate relative transcript levels, which were normalized to the average of two housekeeping genes (indicated in figure legends).

Statistical analysis
The data were analysed using Kruskal–Wallis one-way analysis of variance with Dunn's test to compare specific sample pairs, Mann-Whitney U test, Wilcoxon rank-sum test or a linear model (described below) as detailed in figure legends.

The H3K9me2 immunostaining data shown in Figures 1A, 1C, 4B and Supplemental Figure IB, were analysed for statistical significance using a linear model (fitted in R). The log-transformed H3K9me2/DAPI scores for each cell were expressed as a linear function of the batch (experimental day) and "treatment" groups (e.g. "no surgery" vs. "control" in Figure 1C). In all cases, the model itself was significant ($P<2.2x10^{-16}$) and P-values report where treatment groups were significantly different from controls: HFD vs. SD in Figure 1A ($P<2.2x10^{-16}$), Post injury vs. Control in Figure 1C ($P<2.2x10^{-16}$), A366-treated vs. vehicle in Figure 4B ($P<2.2x10^{-16}$) and ligated vs. internal controls ($P=4.5xe^{-14}$) in Supplemental Figure IB.

To test whether the observed reduction of H3K9me2 in animals fed a high fat versus a standard diet (western blow shown in Figure 1B), we performed one-sided exact Wilcoxon rank-sum tests. We tested against the alternative that H3K9me2 levels were lesser for the high-fat diet than the standard diet respectively (P=0.05). Similar analysis revealed P=0.05 for p-p65 being higher in high fat diet samples compared to controls.

Supplemental Tables

Supplemental Table I: H3K9me2-enriched arteriosclerosis-associated* gene promoters in ex vivo murine VSMCs.

Oprk1
Il17a
Il1rl1
Ctla4
Icos
Smarcal1
Mff
F11r
Crp
Apcs
Il1rn
Grin1
Ttn
Serping1
Bdnf
Il1b
Fgf2
Adora3
Tek
C8b
C8a
Nppb
Nppa
Hs3st1
Cxcl15
Pon1
Zc3hc1
Snca
Reg1
Alox5
Vwf
Klk1
Ctsd
Th
Retn
Nat2
Hmox1
Casp1
Mmp13
Mmp3
Mmp9
Mmp12
Mmp1a
Il18
Lipc
Ccr1
Ccr3
Ccr2
Tcf21
Npm1

Ccl4
Psma6
Serpina3b
Hnrnpc
Myh6
Eif3e
Cyp11b2
Sst
Acat2
Nr3c1
Cybb
Cd40lg
Ace2

*According to The Cardiovascular Disease Portal (https://rgd.mcw.edu/rgdCuration/?module=portal&func=show&name=cardio).

Supplemental Table II: Number of CCAs pooled per *in vivo* RT-qPCR experiment.

Sample	# of CCAs* pooled per experiment	Total number of carotids analysed
L no surgery YFP+	3	12
R no surgery YFP+	3	12
Vehicle L+R no surgery YFP+	2	8
A366 L+R YFP+	2	8
Vehicle R Ligation surgery YFP+	3	12
A366 R Ligation surgery YFP+	3	12
Vehicle L Ligation surgery YFP+	3	12
A366 L Ligation surgery YFP+	3	12
Vehicle L Ligation surgery YFP-ve	1	4
A366 L Ligation surgery YFP-ve	1	4

*CCA, common carotid artery; L, left; R, right.

Supplemental Table III: Primary antibodies

Target antigen	Vendor or Source	Catalog #	Working concentration
G9A	R&D Systems	PP-A8620A-00	2 µg/ml (WB)
H3K9me2	Abcam	ab1220	1 µg/ml (WB), 6.7 µg/ml (ChIP)
H3K9me2 (Alexa Fluor 647)	Abcam	ab203851	10 µg/ml (IF)
IκBα	Cell Signalling	4814	1:1000 (WB concentration not provided)
NFkB-p65	Santa Cruz	sc-372	1 µg/ml (WB, IF)
NFkB-p65	Diagenode	C15310256	1 µl/ChIP (concentration not provided)
NFkB-p65 (Ser536)	Cell Signaling	3033	1:100 (WB, concentration not provided)
cJUN	Abcam	ab31419	1 µg/ml (WB,), 5 ug/ChIP
p-cJUN (Ser63)	Abcam	ab32385	0.1 µg/ml (WB)
p-JNK (Thr183/Tyr185)	Cell Signaling	4668	1:1000 (WB, concentration not provided)
p-ERK1/2 (Thr202/Tyr204)	Cell Signaling	9101	1:1000 (WB, concentration not provided)
p-p38 (Thr180/Tyr182)	Cell Signaling	9211	1:1000 (WB, concentration not provided)
β-Actin	Sigma-Aldrich	A2228	0.1 µg/ml (WB)
β-Tubulin	Cell Signaling	2146	1:1000 (WB, concentration not provided)

*mAb, monoclonal antibody; pAb, polyclonal antibody; †ND, not determined; ‡WB, western blot; §IF, immunofluorescence staining; ChIP, chromatin immunoprecipitation; ‖N/A, not applicable.

Supplemental Table IV: Secondary and IgG control antibodies

Protein	Dilution/ Concentration	Product ID
Anti-rabbit IgG, HRP linked	1:5000	Cell Signaling (#7074)
Anti-mouse IgG, HRP linked	1:5000	Cell Signaling (#7076)
Goat Anti-Rabbit IgG (Alexa Fluor 488)	1:1000	Abcam (#ab150077)
Mouse IgG2a monoclonal [MOPC-173] (Alexa Fluor 647) - Isotype Control	10 µg/ml	Abcam (#ab239458)
Rabbit IgG	6.7 µg/ml (ChIP), 1 µg/ml (IF)	Agilent (X090302-8)

Supplemental Table V: Mouse ChIP-qPCR primer sequences

Gene	Forward 5'-3'	Reverse 5'-3'	Region
Magea2	TTGGTGGACAGGGAAGCTAGGGGA	CGCTCCAGAACAAAATGGCGCAGA	Promoter
Actb	CCACATAGGAGTCCTTCTG	ACCTGTTACTTTGGGAGTGG	Promoter
Mmp2	AGTATCCCTCCAAAGGCAAG	TTCTTCCCACCTCTTCTTCC	Promoter
Mmp3	AAGAAGGTGGACCTAGAAGGAG	CACTGTCATGCAATGGGTAG	Promoter
Mmp9	AAGGCTGAGACCACTGAATG	GTGATTCCATGGTTTGGTG	Promoter
Mmp12	GGCTTTAAGGGAACTTGCAG	ACTGTCATTCATGGGAGCAG	Promoter
Il6	CCCACCCTCCAACAAAGATT	GCTCCAGAGCAGAATGAGCTA	Promoter
Ccl2	CTTCCACTTCCTGGAAACAC	GGCAGCCTTTTATTGTAAGC	Promoter

Supplemental Table VI: Human ChIP-qPCR primer sequences

Gene	Forward 5'-3'	Reverse 5'-3'	Region	Figure
MAGEA2	CCTACACCAACACCATCTTC	AGAATCTCGCTGTCCTCTG	Promoter	5A, 7B
ACTB	CCATTGGCAAGAGCCCGGCT	GACACCCCACGCCAGTTCGG	Promoter	5A, 7B
MMP2	ACGTAGAGGCCAGGAGTAGC	GCCTGAGGAAGTCTGGATG	Promoter	5A
MMP3	GCAAGGATGAGTCAAGCTG	TGTCTCTATGCCTTGCTGTC	Promoter (IL1-responsive element[2], cJUN site[3])	5A, 7B
MMP9	AGAGAGGAGGAGGTGGTGTAAGC	ACCCCACCCCTCCTTGAC	Promoter	5A
MMP12	CAGACGACATGGATCAATTAGC	GTAGGATAGGTGGACGTAGAGGC	Promoter	5A
IL6	CGTGCATGACTTCAGCTTTAC	TGCAGCTTAGGTCGTCATTG	Promoter	5A
CCL2	CCGAGAGGCTGAGACTAACC	CTATGAGCAGCAGGCACAGA	Promoter	5A
IL6	TAGAGCTTCTCTTTCGTTCCCGGT	TGTGTCTTGCGATGCTAAAGGACG	NFkB, cJUN site[4]	5A, 7A, 7B
CCL2	CCTGGAAATCCACAGGATGC	CGAGAGTGCGAGCTTCAG	NFkB, cJUN site[4]	5A, 7A, 7B
MMP3	CATCAAAGGAATGGAGAACC	GACAGAGGTTTCACTATGTTGC	NFkB site[5]	5A, 7A, 7B

Supplemental Table VII: Mouse RT-qPCR primer sequences

Gene	Forward 5'-3'	Reverse 5'-3'	Type
Hprt1	TGGATACAGGCCAGACTTTGTT	CAGATTCAACTTGCGCTCATC	Housekeeping
Hmbs	ACTGGTGGAGTATGGAGTCTCAGATGGC	GCCAGGCTGATGCCCAGGTT	Housekeeping
Ywhaz	CGTTGTAGGAGCCCGTAGGTCAT	TCTGGTTGCGAAGCATTGGG	Housekeeping
Acta2	AGATCTGGCACCACTCTTTC	GTGAGTCACACCATCTCCAG	Contractile VSMC
Myh11	CTGAGGGAGCGATACTTCTC	TGTAGCATGCTTCTGTAGGC	Contractile VSMC
Mmp2	ACTACGATGATGACCGGAAG	TCGGAAGTTCTTGGTGTAGG	MMP
Mmp3	GGATTTGCCAAGACAGAGTG	ATAGGCATGAGCCAAGACTG	MMP
Mmp9	CTCAAGTGGGACCATCATAAC	CGACACCAAACTGGATGAC	MMP
Mmp12	AGCCACACATATCCCAGGAGCA	TCCTGCCTCACATCATACCTCCA	MMP
Il6	TCAATTCCAGAAACCGCTATG	GTCTCCTCTCCGGACTTGTG	Inflammatory
Ccl2	GGCTCAGCCAGATGCAGTTA	CTTCTTTGGGACACCTGCTG	Inflammatory
Cd45	GAGGTGTCTGATGGTGCAAG	TGTATTCCACTAAAGCCTGATGAA	Bone marrow-derived
Pdgfra	GGTCCCAACCTGTAATGAAG	GTAAATGGGACCTGACTTGG	Adventitial fibroblast
Cd31	GTCATGGCCATGGTCGAGTA	CTCCTCGGCATCTTGCTGAA	Endothelial
Sca1	GCTATGGAGTCCCATTTGAG	AGGAAGTCTTCACGTTGACC	Phenotypically switched VSMCs

Supplemental Table VIII: Human RT-qPCR primer sequences

Gene	Forward 5'-3'	Reverse 5'-3'	Type
HMBS	GGTTGTTCACTCCTTGAAGG	TTCTCTGGCAGGGTTTCTAG	Housekeeping
YWHAZ	CGTTGTAGGAGCCCGTAGGTCAT	TCTGGTTGCGAAGCATTGGG	Housekeeping
HPRT1	GTAATTGGTGGAGATGATCTCTCAACT	TGTTTTGCCAGTGTCAATTATATCTTC	Housekeeping
MMP2	ACTACGATGATGACCGGAAG	TCGGAAGTTCTTGGTGTAGG	MMP
MMP3	CTGGGCTATCAGAGGAAATG	CTTATCAGAAATGGCTGCATC	MMP
MMP9	TTGACAGCGACAAGAAGTGG	GCCATTCACGTCGTCCTTAT	MMP
MMP12	CACACCTGACATGAACCGTG	ATGCCAGATCCAGGTCCAAA	MMP
IL6	GGTACATCCTCGACGGCATCT	GTGCCTCTTTGCTGCTTTCAC	Inflammatory
CCL2	AAGATCTCAGTGCAGAGGCTCG	TTGCTTGTCCAGGTGGTCCAT	Inflammatory

JUN	ATCAAGGCGGAGAGGAAGCG	TGAGCATGTTGGCCGTGGAC	AP-1 TF
P65	ATCCCATCTTTGACAATCGTGC	CTGGTCCCGTGAAATACACCTC	NFkB TF
IL1R1	CCTGCTATGATTTTCTCCCAATAAA	AACACAAAAATATCACAGTCAGAGGTAGAC	IL1A receptor

Supplemental Figures

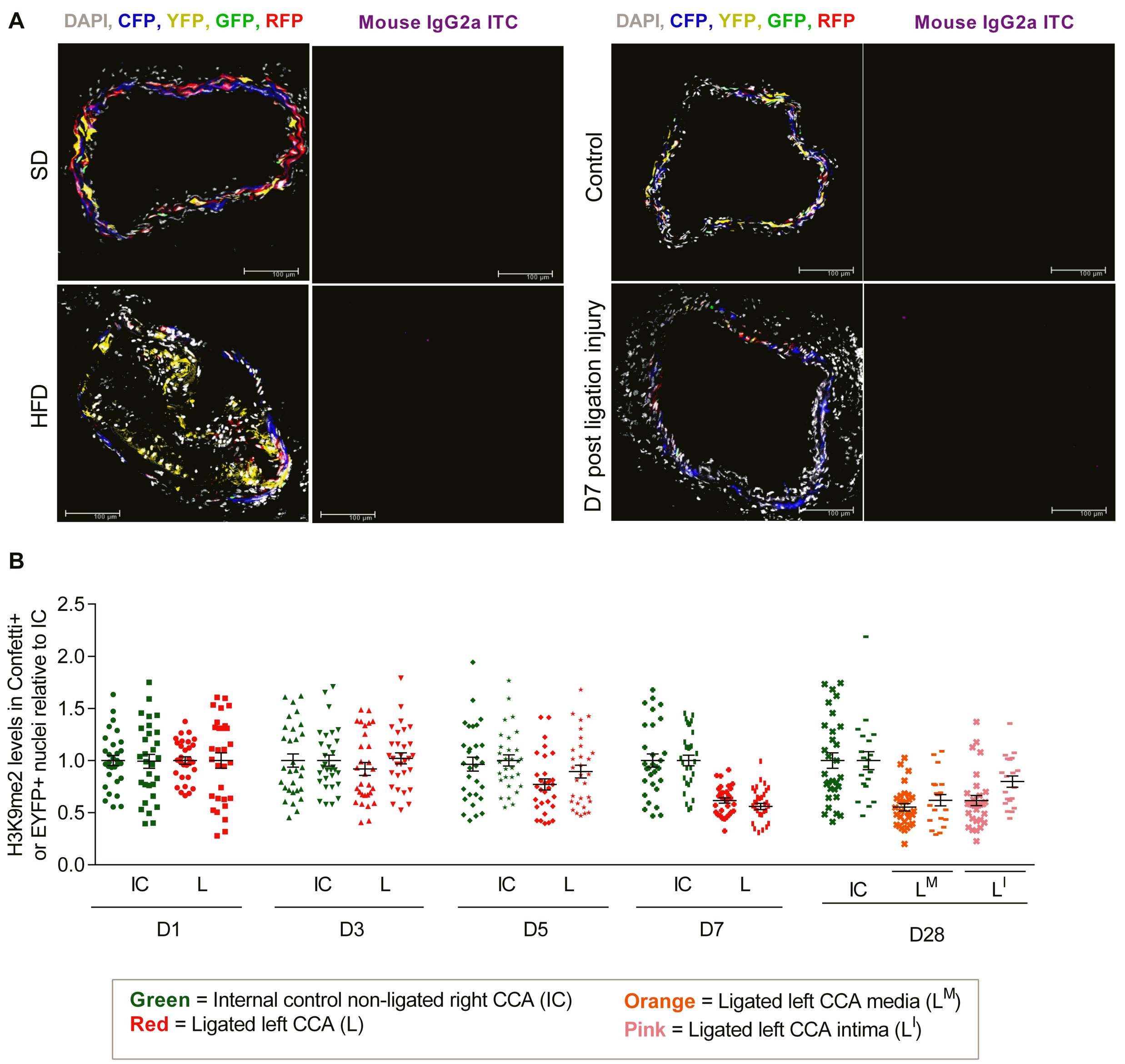

Supplemental Figure I: Global levels of H3K9me2 are reduced in VSMCs upon vascular injury. (A) Control staining with isotype-matched Alexa Fluor 647-conjugated mouse IgG2a of adjacent tissue sections to those shown in main Figure 1A and C. Staining and imaging was done in parallel for H3K9me2 and control staining and image processing was identical. Signals for Confetti-reporter proteins and Alexa Fluor 647 are shown as indicated. Size bars represent 100 μm. **(B)** Box plot showing H3K9me2 signal in VSMC lineage-labelled nuclei (Confetti$^+$ or EYFP$^+$) as determined by immunofluorescence analysis of ligated left (L, red) and non-ligated right internal control (IC, green) CCAs analysed at the indicated day (D) after ligation surgery. At D28, the HK9me2 signal was quantified separately for the media (L^M, orange) and intima (L^I, pink). For H3K9me2 immunofluorescence analyses, each dot shows H3K9me2 signal intensity in one nucleus of a Confetti$^+$ cell relative to the average H3K9me2 signal of the IC of an animal analysed in the same batch (batches are indicated using symbols). n= 2 animals per group. Data for D7 is also included in the data shown in main Figure 1C. Mean (line) and SEM (error bars) are indicated. Ligated samples are significantly different from the IC ($p=1.5 \times 10^{-14}$) as determined by fitting the data to a lineage model (see methods above for details).

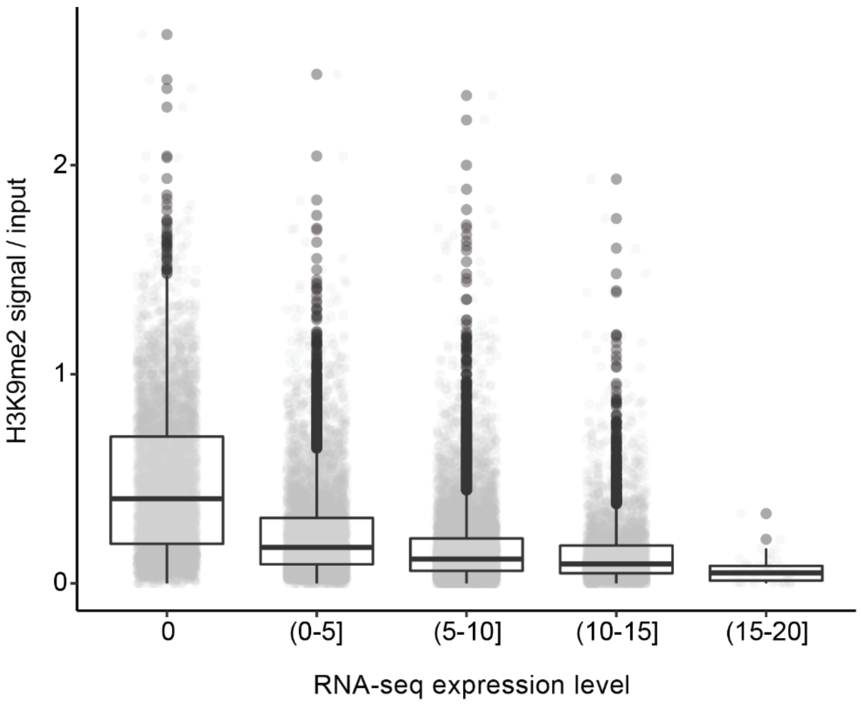

Supplemental Figure II: ChIP-seq for H3K9me2 in ex vivo murine VSMCs. Box plot showing H3K9me2 levels (ratio of H3K9me2 signal / input) within ±1 kb of the transcription start sites of genes against mRNA levels in mVSMCs. Genes with fewer than 20 read counts within ±1 kb of their transcription start site in the input sample were excluded from the analysis. RNA-seq data used in the analysis is publicly available at the Gene Expression Omnibus (accession number GSE117963) and were processed as described[6]. mRNA levels are divided into five bins according to log2-transformed read counts in genes (with pseudocount of 1 added to all genes): genes showing no mRNA expression, over 0 and less than or equal to 5 counts, over 5 and less than or equal to 10 counts, over 10 and less than or equal to 15 counts and over 15 counts and less than or equal to 20 counts. The medians (black line), first and third quartiles (the lower and upper bounds of the boxes, respectively) and the 1.5 interquartile ranges (whiskers) and outliers (dark grey dots) are indicated. Light gray dots show values of individual genes.

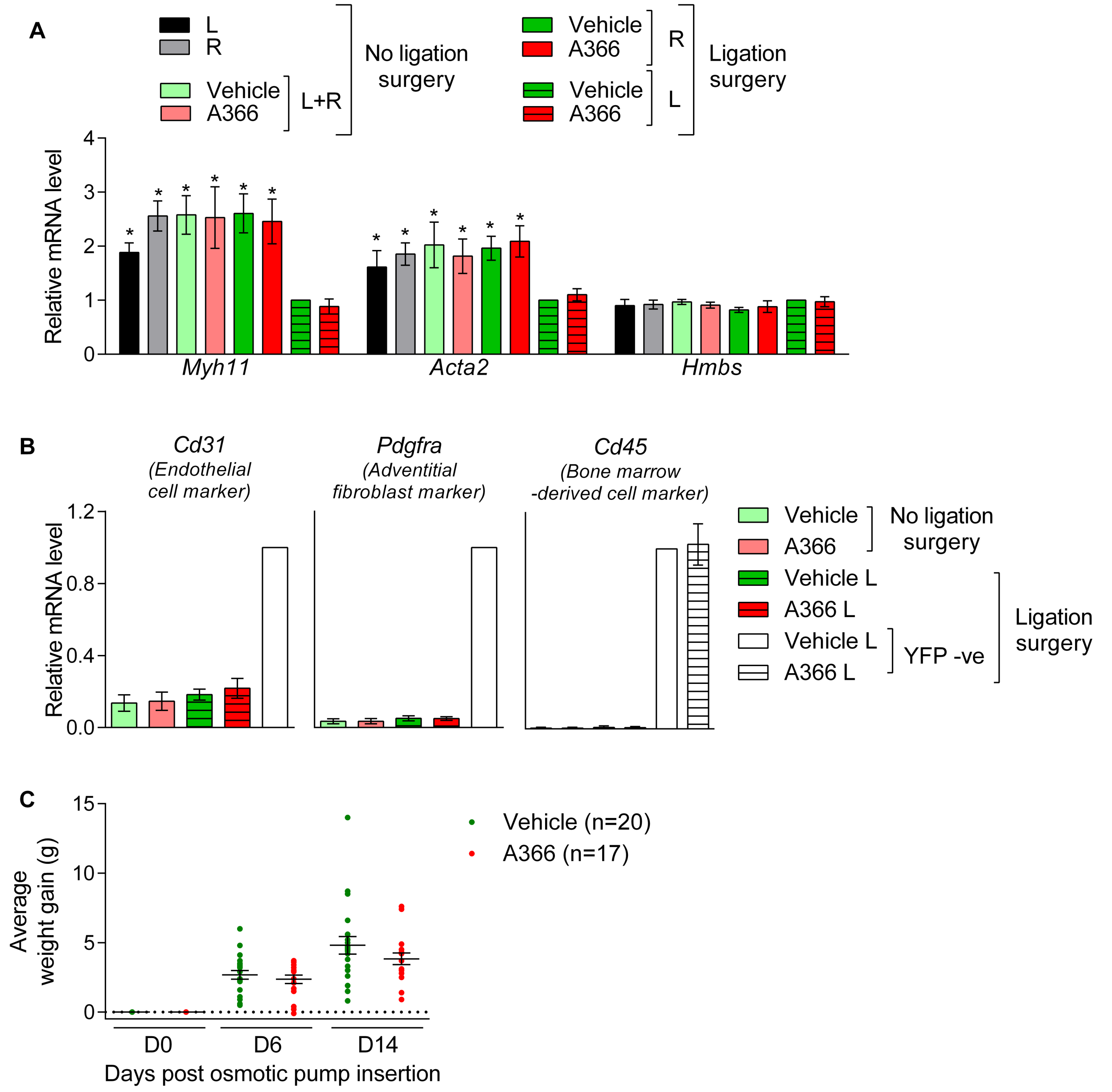

Supplemental Figure III: VSMC-specific upregulation of H3K9me2-target genes after carotid ligation is enhanced by G9A/GLP inhibition. (A) RT-qPCR analysis of *Myh11, Acta2 and Hprt1* transcript levels in EYFP+ cells from the common carotid arteries (CCAs) indicated within the figure panel. Data is normalized to housekeeping genes (*Hprt1* and *Hmbs*) and shown relative to vehicle-control ligated left CCA samples (green striped bars). Graph shows mean±SEM of 4 experiments. *P<0.05 (Kruskal-Wallis, compared to vehicle-treated left ligated CCA). **(B)** RT-qPCR analysis of *Cd31*, *Pdgfra* and *Cd45* transcript levels in EYFP+ cells within the CCAs indicated within the figure panel. Data is normalized to housekeeping genes (*Hprt1* and *Hmbs*) and shown relative to EYFP- (YFP -ve) cells isolated from no surgery control left CCAs (white bars). Graph shows mean±SEM of 3-7 experiments as described in the methods. R= right; L= left. **(C)** Weight gain in animals treated with A366 (n=17) or vehicle control (n=20) relative to the day of osmotic pump insertion. Dots show data for individual animals, horizontal lines show mean and error bars represent SEM.

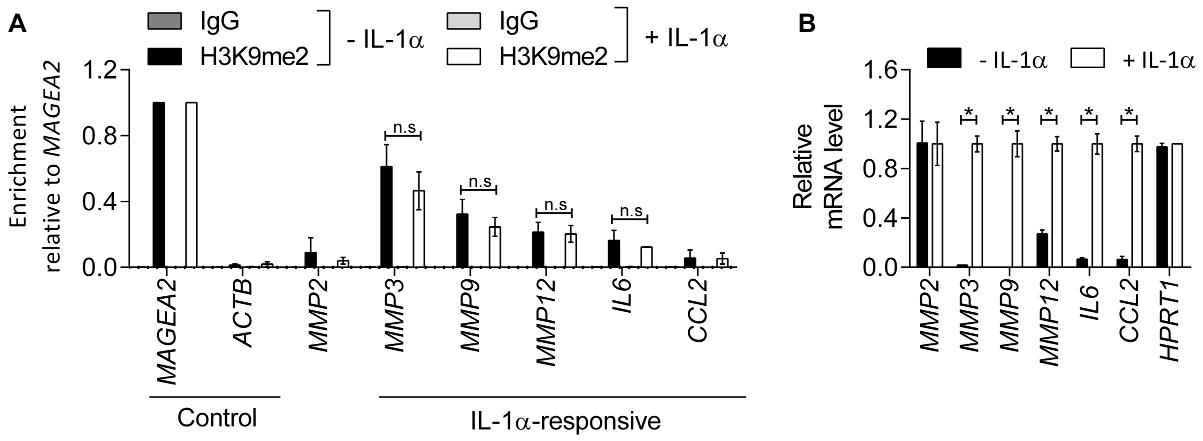

Supplemental Figure IV: H3K9me2 is enriched at a subset of IL-1α responsive gene promoters in human VSMCs. (A) ChIP-qPCR analysis for H3K9me2 in hVSMCs left untreated or treated with IL-1α-treated for 6 hours). Levels of enrichment at *MAGEA2 (*positive control) at *ACTB* (negative control) and the promoters of *MMP2, MMP3*, *MMP9, MMP12, IL6 and CCL2* are compared with signal observed using negative control IgG. Graph show mean±SEM of 4 primary cultures from different individuals. n.s. indicates no statistical significant difference (Kruskal-Wallis). **(B)** RT-qPCR analysis of *MMP2, MMP3*, *MMP9, MMP12, IL6, CCL2* and *HPRT1* transcript levels in untreated control and IL-1α-treated hVSMCs. Expression (mean±SEM in 4 primary cultures from different individuals) is shown relative to IL-1α-treated hVSMCs, normalized to housekeeping genes (*HMBS and HPRT1*). *P<0.05 (Kruskal-Wallis, IL-1α-treated hVSMCs compared to controls).

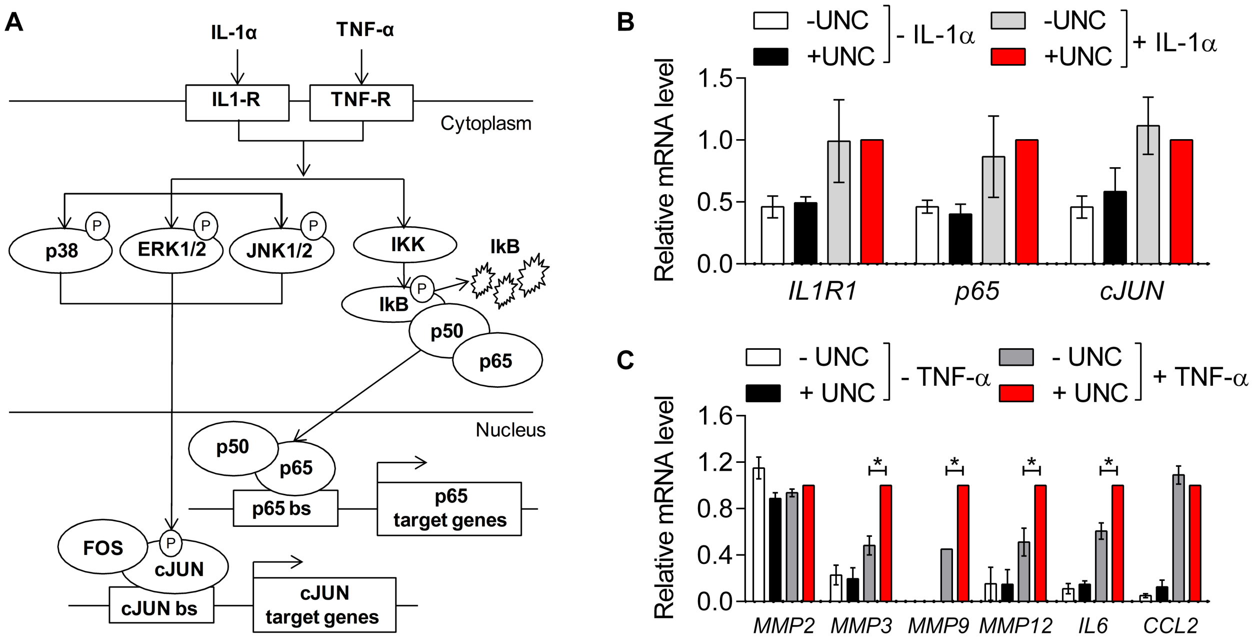

Supplemental Figure V: The effect of G9A/GLP inhibition is shared by IL-1α and TNF-α. (A) Schematic of IL-1α/TNF-α-induced MAPK/NFκB signaling pathways. **(B)** RT-qPCR analysis of *IL1R1*, *p65* and *cJUN* transcript levels in untreated control (white bars), UNC (black bars), IL-1α (grey bars) and UNC plus IL-1α -treated (red bars) hVSMCs. Data is relative to cells treated with UNC plus IL-1α (red bars) and is normalized to housekeeping genes (*HMBS* and *YWHAZ*). Graph shows mean±SEM of 3 primary cultures from different individuals. **(C)** RT-qPCR analysis of *MMP2*, *MMP3*, *MMP9*, *MMP12* and *IL6* and *CCL2* transcript levels in untreated control (white bars), UNC (black), TNF-α (grey) and UNC+TNF-α-treated hVSMCs (red). Data is relative to +UNC +TNF-α (red bars) and normalized to housekeeping genes (*HPRT1* and *HMBS*) (mean±SEM of 3 experiments using primary cultures from different individuals). *P<0.05 (Kruskall-Wallis).

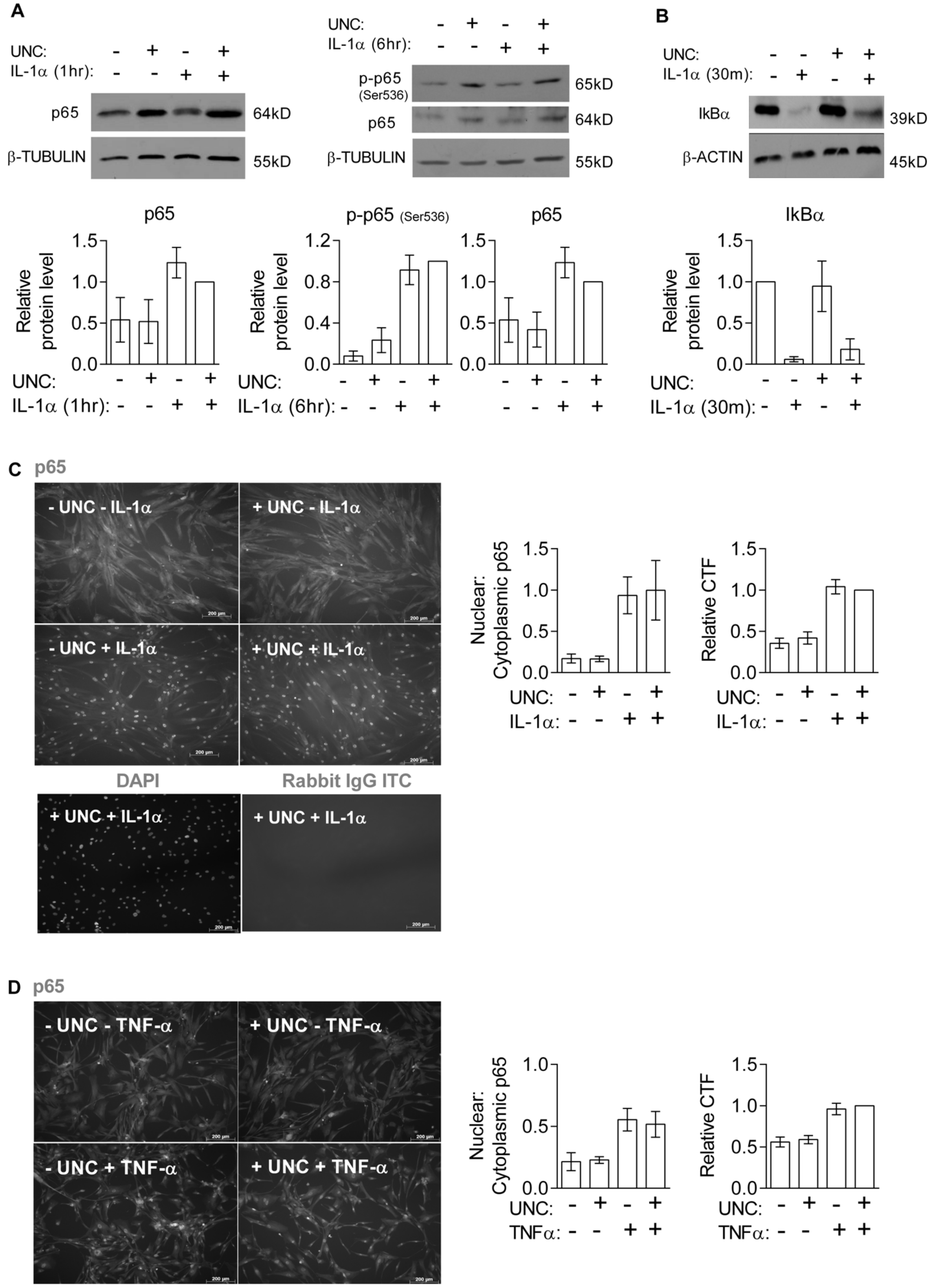

Supplemental Figure VI: G9A/GLP inhibition does not affect NFκB signalling in cultured hVSMCs. (A) Representative western blot and densitometric analysis of p65 and p-p65 (Ser536) in untreated control, UNC, IL-1α and UNC+IL-1α-treated hVSMCs analysed after 1hour (left) or 6 hours (right). Data is relative to cells treated with UNC+IL-1α and is normalized to β-TUBULIN

levels. Graph show mean±SEM of 3 experiments. **(B)** Representative western blot and densitometric analysis of IkBα in untreated control, UNC, IL-1α and UNC+IL-1α-treated hVSMCs analysed after 30 minutes (n=2). **(C, D)** Immunofluorescence staining for p65 (or control rabbit IgG isotype control (ITC)) in untreated control (top left), UNC (top right), IL-1α (C)/ TNF-α (D)(lower left) and UNC+IL-1α (C)/ UNC+TNF-α (D)-treated (lower right) hVSMCs. Signals for p65 (white) are shown. Scale bars, 200 µm. Bar plots show the nuclear:cytoplasmic ratios and corrected total cell fluorescence (CTF) (Means±SEM of 4 (C) or 3 (D) experiments using hVSMCs isolated from independent biopsies). Experiments were done using hVSMCs isolates from different individuals.

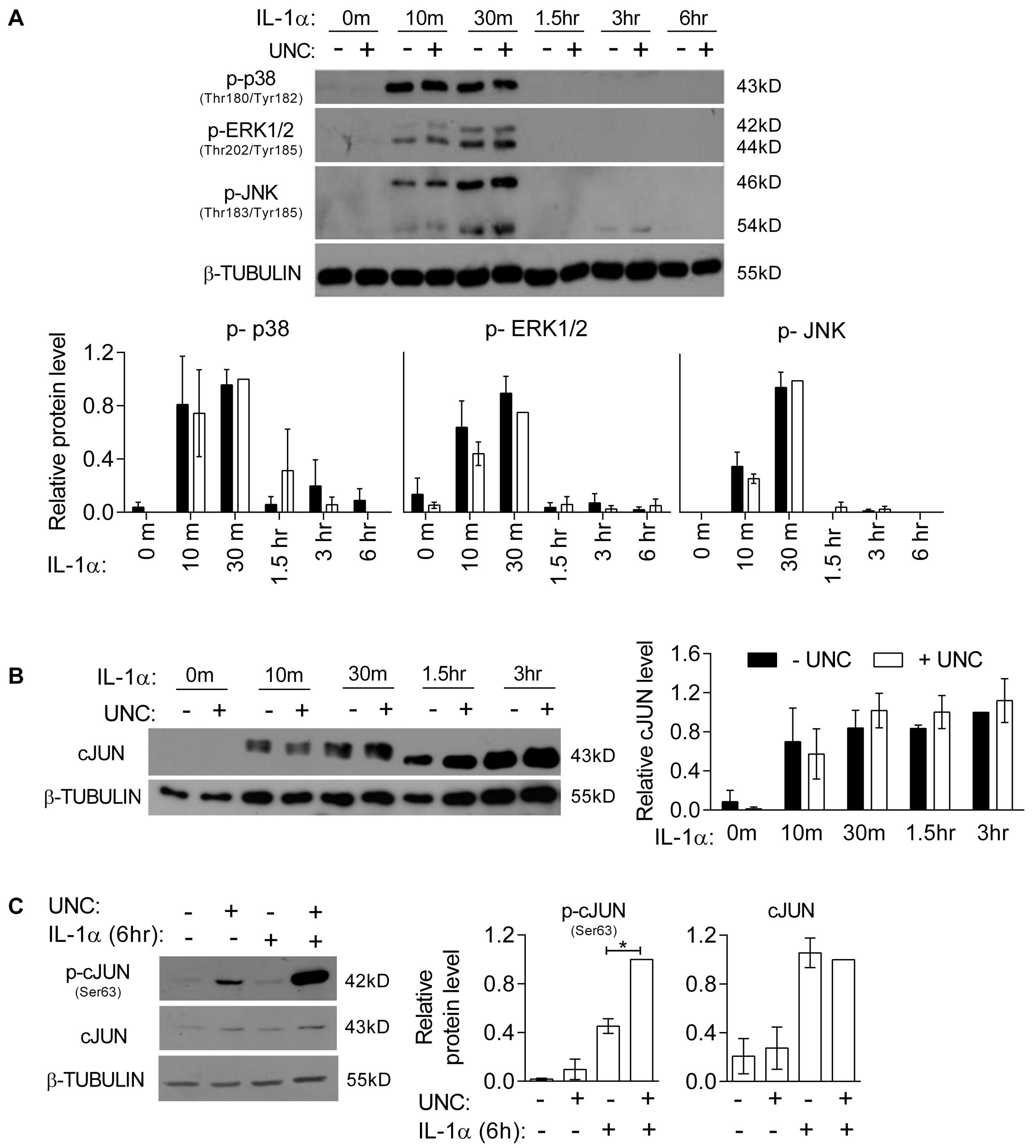

Supplemental Figure VII: G9A/GLP inhibition does not affect cJun signalling in cultured hVSMCs. (A) Representative western blot and densitomeric analysis of p-JNK Thr 183/Tyr 185, p-ERK1/2 Thr 202/Tyr 204, p-p38 Thr 180/Tyr 182 and p-cJUN Ser 63 in untreated controls and hVSMCs treated with IL-1α for 10 mins, 30 mins, 1.5 hr, 3 hr, 6 hr or 24 hrs, without and with prior UNC treatment as indicated. Graph show signals relative to cells treated with UNC and stimulated with IL-1α for 30 mins, normalized to β-TUBULIN levels (mean±SEM of 4 experiments). Each experiment used hVSMCs derived from different individuals. **(B)** Representative western blot and densitometric analysis of total cJUN in untreated controls, and cells treated with UNC and/or IL-1α as indicated. Data is shown relative to cells treated with IL-1α for 3 hrs and is normalized to β-TUBULIN levels. Graph represent means±SEM of 2 experiments. **(C)** Representative western blot and densitometric analysis of p-cJUN (Ser63) and total cJUN in untreated controls, and cells pre-treated with UNC and/or stimulated IL-1α for 6 hours as indicated. Data is normalized to β-TUBULIN levels and shown relative to cells treated with IL-1α. Graph represent means±SEM of 3 experiments. *P<0.05 (Kruskall-Wallis). Experiments were done using hVSMCs isolates from different individuals.

Supplemental references (also included in main text)

1. Thienpont B, Aronsen JM, Robinson EL, Okkenhaug H, Loche E, Ferrini A, Brien P, Alkass K, Tomasso A, Agrawal A, Bergmann O, Sjaastad I, Reik W, Roderick HL. The h3k9 dimethyltransferases ehmt1/2 protect against pathological cardiac hypertrophy. *The Journal of Clinical Investigation*. 2017;127:335-348
2. Quinones S, Buttice G, Kurkinen M. Promoter elements in the transcriptional activation of the human stromelysin-1 gene by the inflammatory cytokine, interleukin 1. *The Biochemical journal*. 1994;302 (Pt 2):471-477
3. Clark IM, Swingler TE, Sampieri CL, Edwards DR. The regulation of matrix metalloproteinases and their inhibitors. *The international journal of biochemistry & cell biology*. 2008;40:1362-1378
4. Sutcliffe AM, Clarke DL, Bradbury DA, Corbett LM, Patel JA, Knox AJ. Transcriptional regulation of monocyte chemotactic protein-1 release by endothelin-1 in human airway smooth muscle cells involves nf-κb and ap-1. *British Journal of Pharmacology*. 2009;157:436-450
5. Borghaei RC, Rawlings PL, Jr., Javadi M, Woloshin J. Nf-kappab binds to a polymorphic repressor element in the mmp-3 promoter. *Biochemical and biophysical research communications*. 2004;316:182-188
6. Dobnikar L, Taylor AL, Chappell J, Oldach P, Harman JL, Oerton E, Dzierzak E, Bennett MR, Spivakov M, Jørgensen HF. Disease-relevant transcriptional signatures identified in individual smooth muscle cells from healthy mouse vessels. *Nature Communications*. 2018;9:4567
