# Supplementary material for: Epigenetic Regulation of Vascular Smooth Muscle Cells by Histone H3 Lysine 9 Dimethylation Attenuates Target Gene-Induction by Inflammatory Signaling
Source: Arterioscler Thromb Vasc Biol. 2019 Aug 22;39(11):2289–302. doi: 10.1161/ATVBAHA.119.312765 (PMC6818986; doi:10.1161/ATVBAHA.119.312765)
Supplement: Supplementary file 2 [file atv-39-2289-s002.pdf]

Major Resources Tables

Animals (all mouse)

Allele information

Allele	Source	Background Strain	MGI
Apoe-/-	Charles River	C57Bl/6	MGI:1857129
Myh11-Cre^{ERT2} (Y-linked transgene)	Offermans lab (Wirth A et al., 2008. Nat Medicine. 14, 64-8)	C57Bl/6	MGI:3819270
Rosa26-Confetti	Hans Clevers (Basak et al., 2014. The EMBO Journal 33, 2057–2068.)	C57Bl/6 (>5 backcrosses)	MGI:4835542
Rosa26-EYFP	Jackson Labs	C57Bl/6	MGI:2449038

Experimental animals

Allele	Vendor or Source	Background Strain	Sex
Apoe-/-; Myh11-CreERt2; Rosa26-Confetti/+	In house breeding	C57Bl/6 (>5 backcrosses)	Male (Myh11-CreERt2 transgene is Y-linked)
Myh11-CreERt2; Rosa26-Confetti/+	In house breeding	C57Bl/6 (>5 backcrosses)	Male (Myh11-CreERt2 transgene is Y-linked)
Myh11-CreERt2; Rosa26-EYFP/EYFP	In house breeding	C57Bl/6	Male (Myh11-CreERt2 transgene is Y-linked)

Animal breeding

	Genotype	Vendor or Source	Background Strain	Other Information
**Parent - Male**	Apoe-/-, Myh11-CreERt2+	In house breeding	C57Bl/6	
**Parent - Female**	Apoe-/-, Rosa26-Confetti/Confetti	In house breeding	C57Bl/6 (>5 backcrosses)	

**Parent - Male**	Myh11-CreERt2+	In house breeding	C57Bl/6	
**Parent - Female**	Rosa26-Confetti/Confetti	In house breeding	C57Bl/6 (>5 backcrosses)	

**Parent - Male**	Myh11-CreERt2+; Rosa26-EYFP/EYFP	In house breeding	C57Bl/6	
**Parent - Female**	Rosa26-EYFP/EYFP	In house breeding	C57Bl/6	

Antibodies

Target antigen	Vendor or Source	Catalog #	Working concentration
G9A	R&D Systems	PP-A8620A-00	2 µg/ml (WB)
H3K9me2	Abcam	ab1220	1 µg/ml (WB), 6.7 µg/ml (ChIP)
H3K9me2 (Alexa Fluor 647)	Abcam	ab203851	10 µg/ml (IF)
IκBα	Cell Signalling	4814	1:1000 (WB concentration not provided)
NFkB-p65	Santa Cruz	sc-372	1 µg/ml (WB, IF)
NFkB-p65	Diagenode	C15310256	1 µl/ChIP (concentration not provided)
NFkB-p65 (Ser536)	Cell Signaling	3033	1:100 (WB, concentration not provided)
cJUN	Abcam	ab31419	1 µg/ml (WB), 5 ug/ChIP
p-cJUN (Ser63)	Abcam	ab32385	0.1 µg/ml (WB)
p-JNK (Thr183/Tyr185)	Cell Signaling	4668	1:1000 (WB, concentration not provided)
p-ERK1/2 (Thr202/Tyr204)	Cell Signaling	9101	1:1000 (WB, concentration not provided)
p-p38 (Thr180/Tyr182)	Cell Signaling	9211	1:1000 (WB, concentration not provided)
β-Actin	Sigma-Aldrich	A2228	0.1 µg/ml (WB)
β-Tubulin	Cell Signaling	2146	1:1000 (WB, concentration not provided)
Anti-rabbit IgG, HRP linked	Cell Signaling	7074	1:5000 (WB, concentration not provided)
Anti-mouse IgG, HRP linked	Cell Signaling	7076	1:5000 (WB, concentration not provided)
Goat Anti-Rabbit IgG (Alexa Fluor 488)	Abcam	ab150077	1:1000 (concentration not provided)
Mouse IgG2a [MOPC-173] (Alexa Fluor® 647) - Isotype Control	Abcam	ab239458	10 µg/ml (IF)
Rabbit IgG	Agilent	X090302-8	6.7 µg/ml (ChIP), 1 µg/ml (IF)

Cultured Cells

Name	Vendor or Source	Sex (F, M, or unknown)
WT murine VSMCs	Primary culture setup from C57Bl/6 animals purchased from Charles River)	Males
Human VSMC	Primary culture (used in Figures 5B, 5C, 5E, 5F)	M (20 years)
Human VSMC	Primary culture (used in Figures 5B, 5C, 5E, 5F)	F (33 years)
Human VSMC	Primary culture (used in Figures 5B, 5C, 5E, 5F and Supplemental Figure 6B)	M (56 years)

Human VSMC	Primary culture (used in Figures 5B, 5C, 5E, 5F and Supplemental Figure 4A, 4B and 5B)	F (64 years)
Human VSMC	Primary culture (used in Figures 5C, 5D and Supplemental Figures and 6A, 7A, 7B and 7C)	M (32 years)
Human VSMC	Primary culture (used in Figures 5A, 5C, 5D, 6C and Supplemental Figures 6A, 6C, 7A and 7C)	M (44 years)
Human VSMC	Primary culture (used in Figures 5A, 5C, 5D, 6B, 6C and Supplemental Figures 6A)	F (71 years)
Human VSMC	Primary culture (used in Figures 5A, 5C and Supplemental Figures 7A, 7B and 7C)	F (77 years)
Human VSMC	Primary culture (used in Figure 5A, 5C and Supplemental Figures 4A, 4B and 7A)	M (53 years)
Human VSMC	Primary culture (used in Figure 5A and Supplemental Figures 4A and 4B)	M (66 years)
Human VSMC	Primary culture (used in Figure 5A and Supplemental Figures 4A and 4B)	M (78 years)
Human VSMC	Primary culture (used in Figures 6B, 6D and Supplemental Figures 5B, 5C, 6C and 6D)	M (41 years)
Human VSMC	Primary culture (used in Figures 6B, 6D and Supplemental Figure 5B 5C, 6C and 6D)	F (53 years)
Human VSMC	Primary culture (used in Figures 5C, 6B, 6D and Supplemental Figures 5C and 6D)	M (45 years)
Human VSMC	Primary culture (used in Figures 6C, 6D and Supplemental Figure 6C)	M (69 years)
Human VSMC	Primary culture (used in Figure 6C and Supplemental Figure 6B)	M (77 years)

Reagents

Name	Vendor or Source		Working Concentration
SP600125	Abcam	ab120065	10 μM
UNC0638	Tocris/Sigma	4343/U4885	1 μM
A366	Tocris	5163	1 μM
IL-1 α	Peprotech	210-10	2 ng/ml
TNF-α	Peprotech	300-01A	90 ng/ml
siRNA (G9A)	Santa cruz	sc-43777	90 ng/ml
siRNA (Control)	Santa cruz	sc-37007	90 ng/ml
HiPerfect	Qiagen	301704	NA
DQ Gelatin	Thermo Fisher	D12054	50 μg/ml (1 ml per 9.5 cm^2)
Osmotic pump	Alzet	2002	NA
iDeal ChIP-seq Kit for Transcription Factors	Diagenode	c01010054	NA
